# Earthquake source characterization by machine learning algorithms applied to acoustic signals

**DOI:** 10.1038/s41598-021-02483-w

**Published:** 2021-11-29

**Authors:** Bernabe Gomez, Usama Kadri

**Affiliations:** School of Mathematics, Cardiff University, Senghennydd Road, Cardiff, CF24 4AG UK

**Keywords:** Natural hazards, Acoustics

## Abstract

Underwater seismic events generate acoustic radiation (such as acoustic-gravity waves), that carries information about the source and can travel long distances before dissipating. Effective early warning, emergency response, and information dissemination for earthquakes and tsunamis require a rapid characterisation of the fault properties: geometry and dynamics. In this work, we analysed hydrophone recordings of 201 earthquakes, located in the Pacific and the Indian Ocean, by employing acoustic signal processing and classification methods. The analysis allows identifying the type of earthquake (i.e. slip type, magnitude) and provides near real-time estimation of the effective properties of the fault dynamics and geometry. The results were compared against values reported by the Harvard Global Centroid Moment Tensor catalog (gCMT), revealing statistical significance between the extracted acoustic properties used to feed machine learning algorithms and the predicted slip and magnitude values.

## Introduction

Underwater seismic events can produce very long compression-type waves, known as acoustic-gravity waves (AGWs), that propagate in the water layer travelling long distances with almost no attenuation^1^ and can be recorded by distant hydrophones. This property of AGWs allows them to carry information on the sound source^1,2^. The classification and characterisation of such information are important for the assessment of potential Tsunamis. In order to characterise tectonic events, the source dimensions, dynamics and moment magnitude need to be estimated, which can be approached by automated underwater acoustic signal processing methods.

Gomez and Kadri^3^ proposed an inverse problem model which calculates the effective fault dimensions and vertical uplift speed and duration induced by underwater earthquakes, using slender fault theory^1^. However, this model can be applied only when the slip direction is vertical. Thus, there is a need to identify the slip direction prior to applying the model in real-time. To this end, we consider Anderson’s faulting theory (1905)^4^ where the equations of stress produced over the fault planes in an earthquake are analysed and earthquakes are divided into three classes depending on the faulting type: wrench (when the greatest pressure is in the horizontal plane), normal, and reversed. This 3-type classification has been widely accepted and used in the literature^5,6^. Nevertheless, previous studies indicate that tectonic events can be further grouped in only two types, dip-slip and strike-slip, depending on the direction of the dominant motion component^7^. Thus, allowing the discrimination of events with significant vertical slip, which can be performed by machine learning (ML) techniques. The application of ML algorithms to acoustic signals in the ocean has had increasing notability in recent years, such as the classification of vessels^8^, earthquakes^9,10^, tsunamigenic events^11^, underwater explosions^9,10^ and marine life^12^, to name a few. An important input parameter for the inverse problem model is earthquake magnitude. Relations between the maximum amplitude of T-phase waves, the earthquake’s energy that propagates through the SOFAR channel (minimum sound speed channel in the ocean)^13,14^, and earthquake size^15^, or correlations between T-phase power level and seismic moment have been developed in previous studies^15,16^. Other approaches are based on estimating tectonic event magnitudes employing ML techniques to seismic recordings^17–19^. In this study, relationships between acoustic waves characteristics and underwater seismic event magnitudes are approached using ML regression algorithms. Once the earthquake moment magnitude is obtained, relations between fault rupture dimensions and earthquake magnitude can be used to estimate seismic hazards^20^, where earthquakes are mapped into effective slender geometries^21^, that can be verified from aftershock distributions^22^.

**Figure 1:**
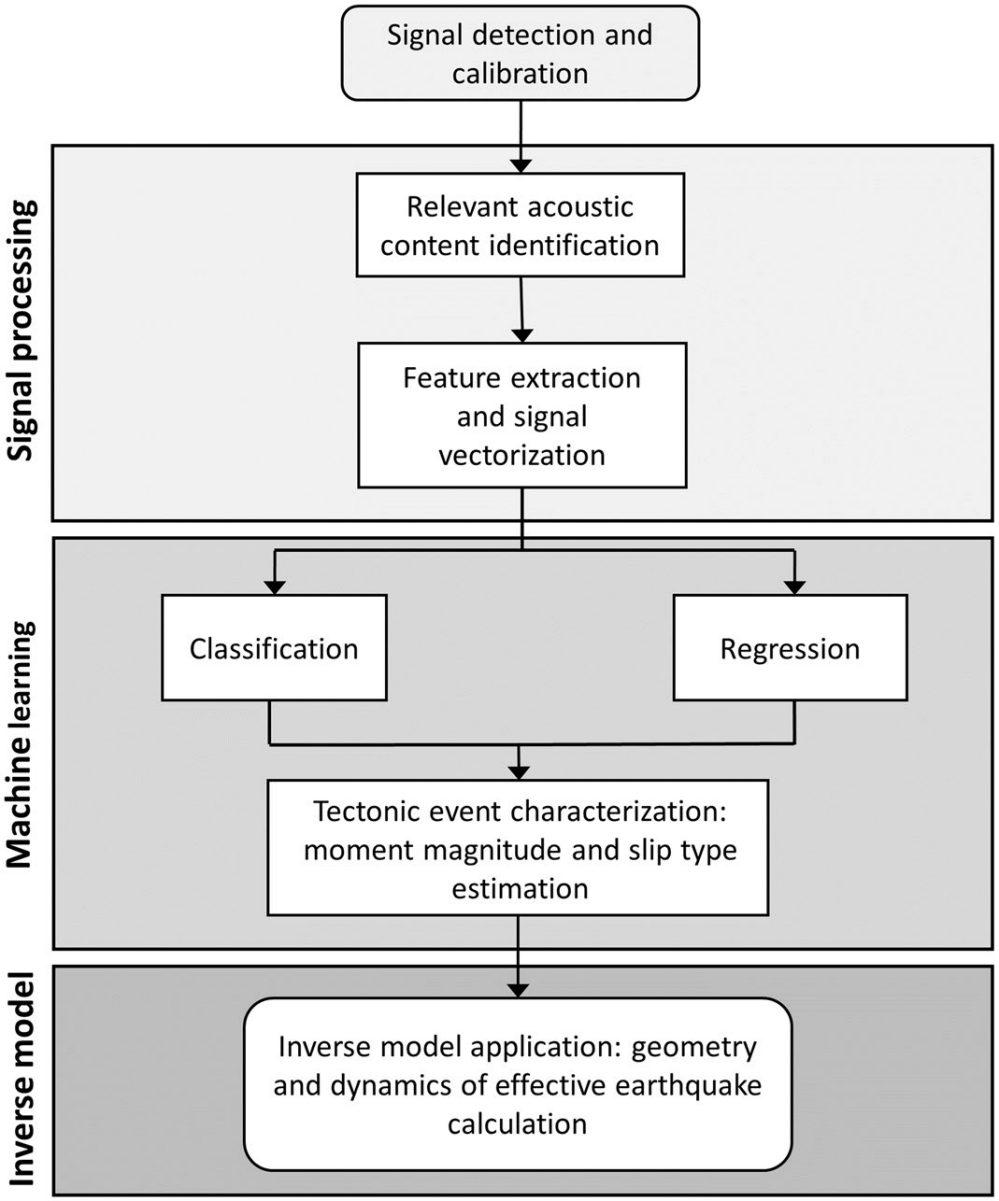
Flow chart for the methodology of tectonic event characterization from acoustic recordings analysis.

Comprehensive Nuclear-Test-Ban Treaty Organization (CTBTO) acoustic recordings have been previously studied for identification and detection of T-waves^23,24^ or classification and regression of sound signals related to tectonic events^9,10,25^. In this work, we analyse 201 acoustic signals related to submarine tectonic events with magnitudes ranging from 5 to 9.1 Mw and different associated slip types. The signals were recorded by three different CTBTO^26,27^ hydro-acoustic stations located in the Indian and the Pacific Oceans. To characterise tectonic events, first, the associated pressure disturbance is identified in the hydrophone recordings and feature extraction is performed (Fig. 1). Feature vectors serve as input to ML algorithms that perform classification of the slip type (existence of significant vertical motion component) and assessment of the magnitude of the event. This information is then used to feed an inverse problem model for acoustic waves that calculates the effective geometry and dynamics of the fault^3^.

Due to the limited size of the dataset there is room for improvement in the accuracy results. Thus, this work is a proof of concept that ML algorithms coupled with an inverse model for acoustic waves can provide early characterizations for tectonic events.

## Results

ML algorithms were applied to the acoustic signal properties to estimate two main characteristics of the studied tectonic events: slip type (qualitative) and moment magnitude (quantitative, Mw). Thus, two types of ML algorithms were considered: classification (slip type) and regression (magnitude).

### Slip type classification

The primary objective of the classification is to identify the existence of significant vertical motion components in the studied tectonic events. In addition, as a secondary objective, we study the characterization of the type of vertical motion related to the studied earthquakes. Two classification approaches were taken to identify the slip type associated with the tectonic events that generated the acoustic signals composing the dataset: binary and multi-class.

#### Binary classification

The dataset is divided into two classes. The first class is composed of signals related to tectonic events with vertical motion components (mostly dip-slip), whereas the second is composed of events with relatively small or no vertical motion components (mostly strike-slip). The differentiation between vertical and horizontal events is essential when applying the inverse problem model developed by Gomez and Kadri^3^, which is designed to work for vertical fault displacements. The division of the dataset was made based on source faulting solutions provided by the global CMT catalog^28,29^, resulting in a set distribution of 86 strike-slip earthquakes, which are considered to have mainly horizontal motion component (42.79%) and 115 earthquakes with relevant vertical motion component (57.21%). In that sense we insure that the dataset is balanced between events with significant vertical motion components and mainly horizontal slip events.

**Figure 2:**
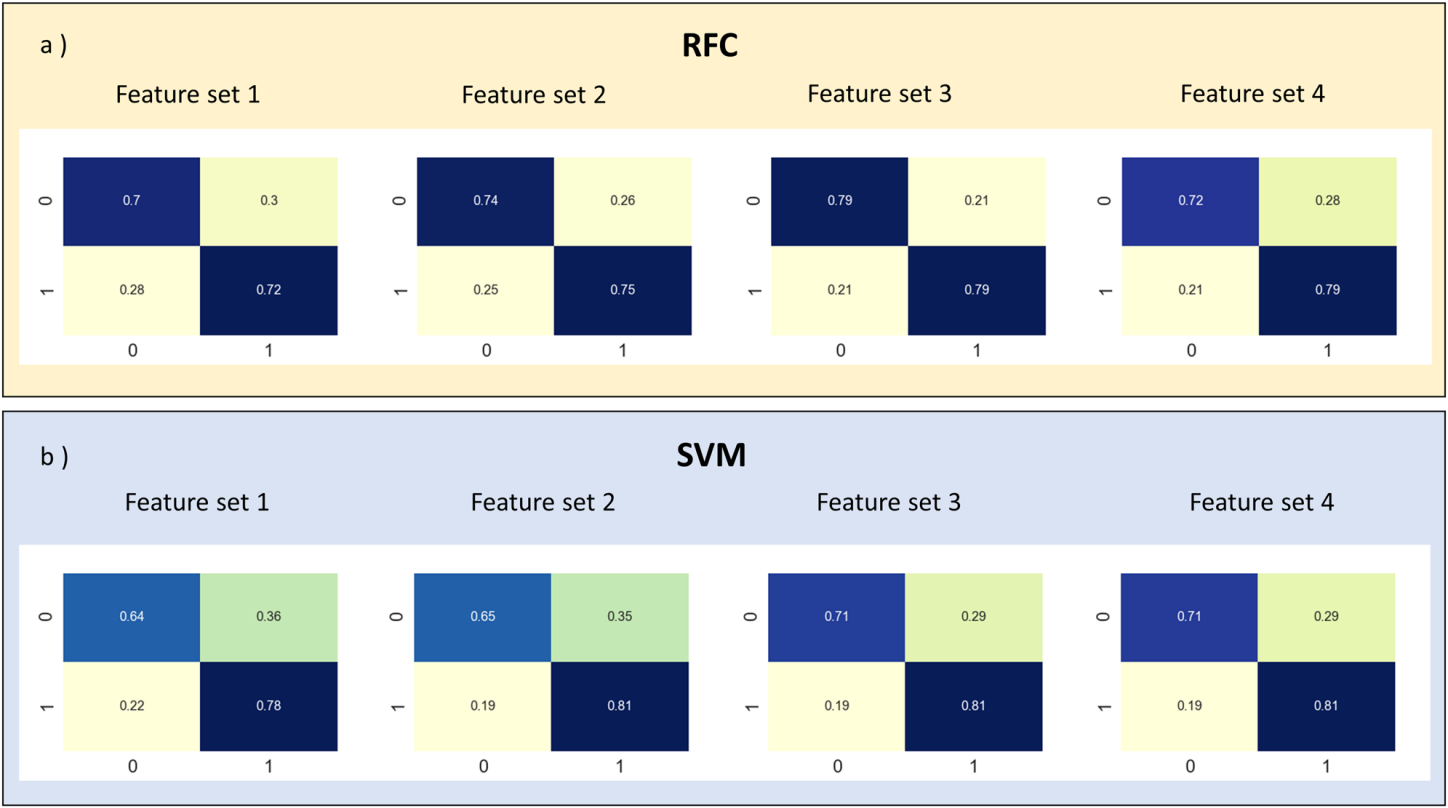
Binary classification confusion matrices for the considered feature sets and classification algorithms. ‘0’ stands for events classified as mainly horizontal slip motion and ‘1’ for events with relevant vertical motion component. (**a**) Normalised absolute errors for the RFC application along with 10-fold validation scheme. (**b**) Normalised absolute errors for the SVM application along with 10-fold validation scheme.

In order to characterize the acoustic signals, we applied four different methodologies for feature extraction that are described in detail in the “Signal vectorization (feature extraction)” subsection in the “Methods” section. In addition, two classification algorithms were applied along with each feature extraction methodology used on the dataset: Random Forest Classifier (RFC) and Support Vector Machines (SVM). In order to test the ML algorithms, we used 10-fold validation technique and 5-fold hyper-parameter grid search, for more details see the ‘k-fold and grid search’ subsection in the “Sensitivity analysis” section in the Supplementary materials. Results obtained for every fold were averaged to provide final accuracy and standard deviation estimates, see Table 1. Moreover, confusion matrices were computed in order to provide classification accuracy for each considered slip type, see Fig. 2.

**Table 1:** Accuracy and standard deviation (%) for binary classification using SVM and RFC on four different sets of features.

Feature set	SVM accuracy (%)	RFC accuracy (%)
1	71.62 ± 11.67	71.12 ± 9.75
2	74.10 ± 10.64	75.60 ± 10.64
3	76.64 ± 8.86	78.10 ± 8.74
4	76.12 ± 10.76	73.62 ± 10.76

In all cases, there is a higher performance of the ML algorithms for the identification of ‘vertical earthquakes’ (‘1’) compared to the identification of ‘horizontal earthquakes’ (‘0’), see Fig. 2. This behaviour could be associated with the higher availability of ‘vertical earthquakes’ in the studied dataset. The overall accuracy of both ML algorithms applied to each feature set is over 70% for every considered scenario. Both ML algorithms show similar accuracy for binary classification of the dataset.

#### Multi-class classification

Secondary results are presented in this subsection, where the relations between earthquake slip types and their associated acoustic signals are further studied. Here, the dataset was split into three classes^4^ based on the source faulting solutions provided by the global CMT catalog^28,29^. The classes are strike-slip (‘0’), thrust or reverse (‘1’) and normal (‘2’). Thus, the ‘vertical’ class, defined in the previous subsection, was further subdivided into two classes: thrust and normal. This classification led to a dataset distribution composed of 86 strike-slip (42.8%), 62 reverse (30.8%) and 53 normal earthquakes (26.4%). The signal vectorization techniques used in binary classification have been utilised in the section. RFC and SVM were applied, where a ‘One-versus-all’^30^ technique was used, generating as many binary classifiers as label types and testing every class against the rest. The resulting classification accuracy, standard deviations and confusion matrices are provided in the Supplementary materials, ‘Multi-class classification’ section.

The application of RFC to the feature set ‘3’, where only features obtained by applying the DWT are considered, led to the highest observed accuracy results, with an average accuracy for 10-fold validation technique of 64.14% and 8.99% standard deviation, see ‘Multi-class classification’ section in the supplementary materials. The normalized confusion matrices suggest that the algorithms classified the majority of the strike-slip and thrust earthquakes accurately (> 70%), though failed to identify most of the normal earthquakes (< 30%). The low classification accuracy of normal slip type events is potentially influenced by a low presence of acoustic signals related to this type of earthquake in the dataset, leading to an imbalanced dataset^31^. Nevertheless, the overall classification accuracy (> 60%) for most of the cases reveals statistical significance between the features and the slip types.

### Magnitude regression

**Figure 3:**
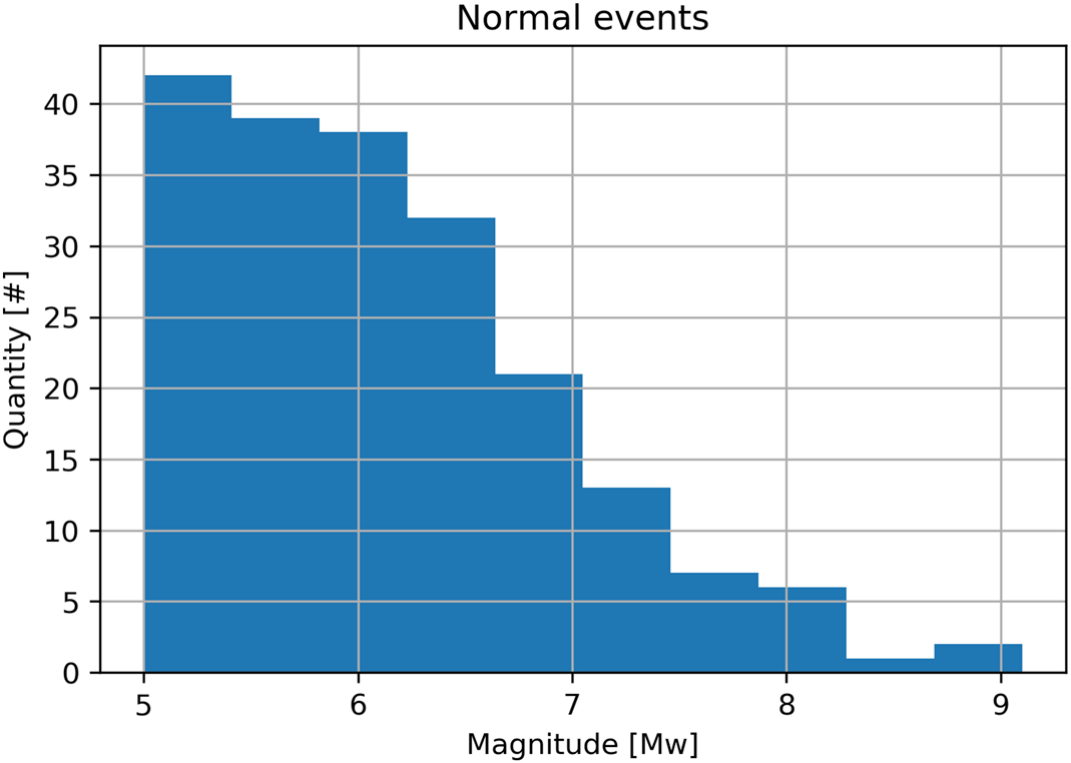
Moment magnitude distribution for the studied dataset.

In this section, we estimate the magnitude of tectonic events by analysing acoustic signals and applying ML algorithms. Support Vector regressor (SVR) and Random Forest regressor (RFR) were applied to each of the four different feature sets extracted from the recorded signals that compose the dataset. Additionally, we used 10-fold validation technique and 5-fold hyper-parameter grid search. The magnitudes (Mw) of the events were obtained from the global CMT catalog^28,29^, see Fig. 3. Note that since the frequency of occurrence of an Earthquake drops logarithmically with the magnitude, having a perfectly balanced dataset is rather challenging. The magnitude frequency for each type of earthquake is graphically analysed in the Supplementary materials, ‘Multi-class classification’ section.

The sum of squared errors (SSE) associated with the results delivered by the ML regression algorithms is calculated by

1 $$\begin{aligned} SSE = \sum _{i=1}^{n} (y_i-f(x_i))^2, \end{aligned}$$

where *n* is the size of the test set, $y_i$ are the actual values in the test set and $f(x_i)$ are the predictions made by the ML algorithms.

The calculated SSE values for each fold were averaged to provide final estimates for the performance of the regression algorithms, see Table 2. The SSE, in Eq. (1), was applied using the average of the training set as predictor $f(x_i)$, resulting in 13.61, which is about twice the SSE values obtained using the ML models predictions presented in Table 2. This indicates that the model delivers more accurate predictions than the mean value of the training set. Additionally, the R-squared ($R^2$) estimator, which represents the part of the variance for a dependent variable that is explained by the independent variables in a regression algorithm, is computed by

2 $$\begin{aligned} R^2 = 1- S_{res}/ S_{tot}, \end{aligned}$$

where $S_{res}$ is the sum of squares of the residual errors and $S_{tot}$ is the total sum of the errors.

**Table 2:** Calculated SSE for the algorithm estimations against the actual values and $R^2$ score for each considered ML algorithm and feature set.

Model	Set 1		Set 2		Set 3		Set 4	
	SSE model	$R^2$ score	SSE model	$R^2$ score	SSE model	$R^2$ score	SSE model	$R^2$ score
SVR	5.94	0.51	6.07	0.49	7.19	0.42	7.72	0.40
RFR	6.30	0.48	6.54	0.45	7.18	0.42	7.19	0.41

The lowest errors were obtained by applying SVR to the set of features ’2’, which is composed of a combination of temporal, statistic and cepstral features. The observed $R^2$ scores lie around 0.5, suggesting that the regression algorithms in combination with the extracted features were able to explain some relations between the extracted signal features and the corresponding tectonic event magnitudes, see Table 2. The calculated and actual values for the magnitudes of the tectonic events are graphically compared in the ‘Regression accuracy distribution’ subsection in the ‘Sensitivity analysis’ section in the ‘Supplementary materials’. It was observed that the estimations related to magnitude extremes of the dataset ($Mw<6$ and $Mw>8$) have higher associated errors.

### Earthquake case studies

To demonstrate the applicability of the developed methodology, we have chosen five real case scenarios independent from the dataset used to train the ML algorithms:

- 16th February 2015 (39.78N 143.22E).
- 14th March 2012 (40.88N, 144.93E).
- 25th October 2013 (27.17N, 144.66E).
- 21st December 2010 (27.10N, 143.76E).
- 29th September 2009 (-15.13N, -171.97E).

The selected earthquakes have a wide range of magnitudes (6.7 to 8.1 Mw). Stronger earthquakes were not considered due to the small number of available recordings associated with them. The studied earthquakes in this section are located in the Pacific ocean and their estimated properties are compared against data extracted from the gCMT^28,29^. Additionally, reports released by the National Oceanic and Atmospheric Administration (NOAA) show that the selected earthquakes triggered tsunamis.

The acoustic signals emitted by the earthquakes were recorded by the IMS hydrophone station ‘HA11’ situated in the middle of the Pacific ocean and deployed by CTBTO. These earthquake scenarios were chosen as they do not have abrupt bathymetry changes in the paths between their epicentres and the ‘HA11’ hydrophone station, reducing the inverse problem uncertainties. The process taken for retrieving the type of slip, magnitude and effective properties of a tectonic event is detailed in this section for the case of the introduced event 16/02/2015. Then, the results obtained for the remaining four earthquakes are provided in Tables 5 and 6.

The gCMT^28,29^ reported an earthquake of magnitude 6.7 Mw and coordinates Lat=39.78, Lon=143.22 (Offshore, near the east coast of Honshu), that occurred on 16/02/2015 at 23:06 UTC. The epicentre location is about 2 km deep underwater, with a hypocenter situated 22.2 km under the ground-water interface. The half duration of the event is 5.7 seconds that was catalogued as a thrust earthquake with slip angles: strike = 182° and 26°; dip = 18° and 73°; slip = 68° and 97°. Moreover, the studied event triggered a small tsunami with a wave height of 20 cm, recorded along the coast of Iwate (https://www.ngdc.noaa.gov/hazel/view/hazards/tsunami/event-search).

**Figure 4:**
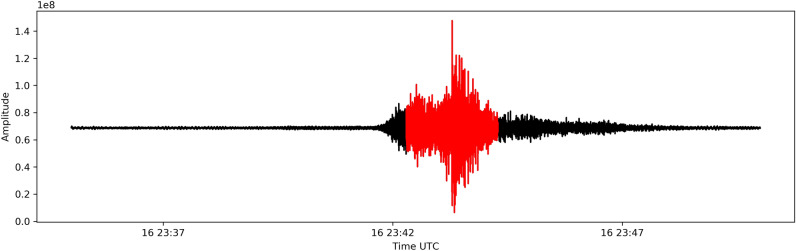
Signal related to the studied tectonic event recorded by the *‘H11N1’* hydrophone. In red highlighted relevant acoustic disturbance.

The distance between the analysed recording hydrophone and the epicentre of the tectonic event is approximately 3200 km, resulting in a calculated travel time for the acoustic waves of 35 mins before reaching *‘H11N1’*, see Fig. 4. The extracted hydrophone recordings, displayed in Fig. 4, begin at 23:35 UTC and end at 23:50 UTC, the arrival of the disturbance is identified around 23:42 UTC, see Fig. 4.

The acoustic disturbance was isolated and the four proposed sets of features extracted. The dataset composed of 201 acoustic signal recordings associated with tectonic events was used to train the classification and regression algorithms, which estimated the slip type of the studied event with 100% accuracy and the magnitude with an associated error lower than 5%, see Tables 3 and 4.

**Table 3:** Classification results for the study case using four different feature sets. For the 3-type classification ‘0’ stands for horizontal, ‘1’ for thrust and ‘2’ for normal.

	Binary				Multi-class			
	1st	2nd	3rd	4th	1st	2nd	3rd	4th
SVM	1	1	1	1	1	1	1	1
RFC	1	1	1	1	1	1	1	1

For the binary classification ‘0’ represents horizontal, ‘1’ for vertical.

**Table 4:** Regression results [Mw] for both considered algorithms applied to the four feature sets extracted from the study case signal.

	1st	2nd	3rd	4th
SVR	6.72	6.71	6.79	6.49
RFR	6.79	6.69	6.78	6.40

The earthquake’s type of motion and magnitude estimations delivered by the ML algorithms, reported in Tables 3 and 4 , are important input parameters for the inverse problem model, see ‘Inverse problem model’ section. When the earthquake is classified vertical (binary classification) the inverse problem model can be applied. The potential ranges for the effective fault size and dynamics are calculated by using empirical relations that relate the magnitude of the event with the effective rupture length, width, and ground surface displacement^20^. These ranges are finally fed into the inverse problem model.

**Figure 5:**
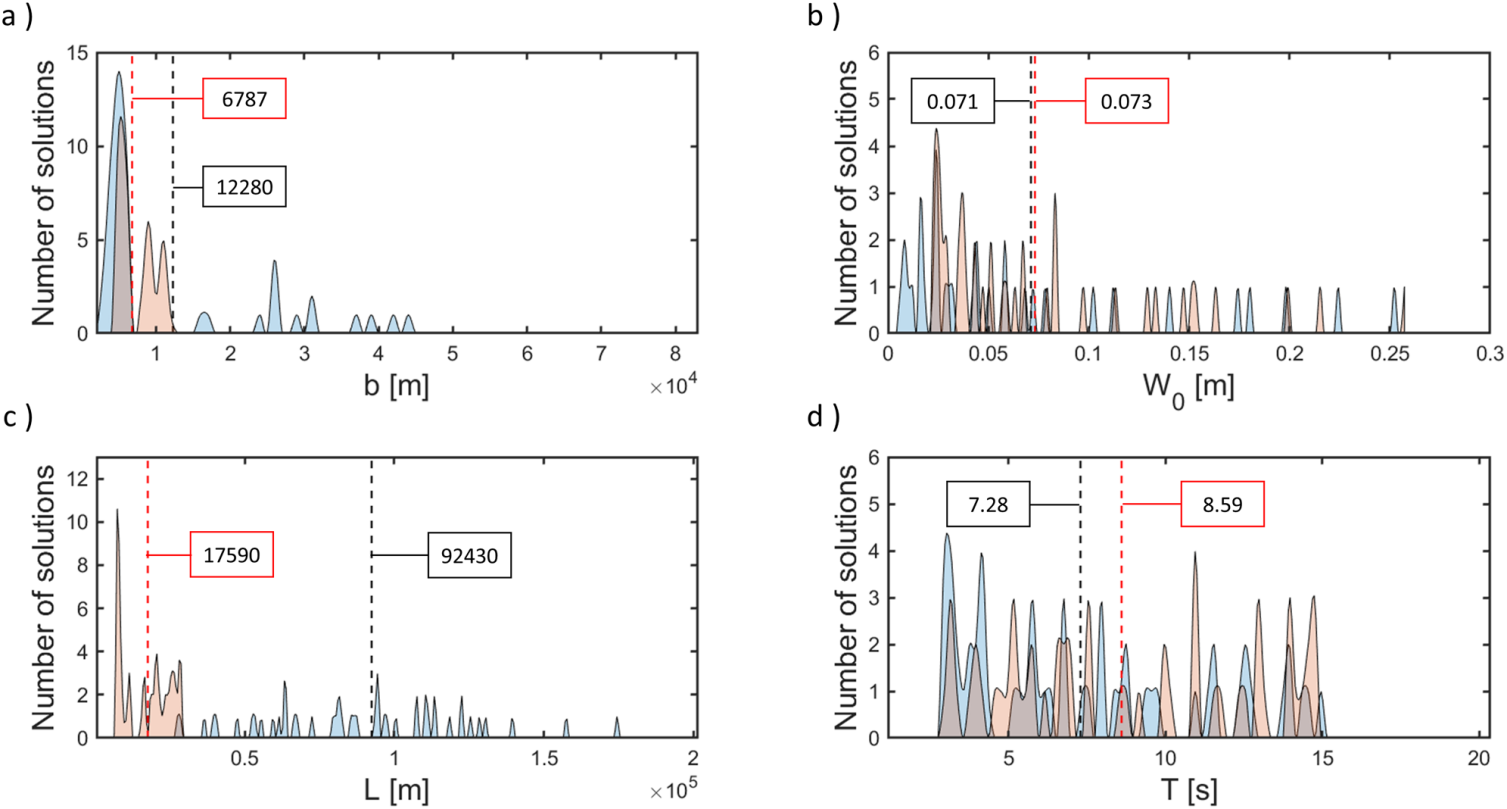
Probability density functions for the effective slender fault characteristics calculated by the inverse problem model. In blue, the results were calculated without taking into account the moment magnitude of the studied tectonic event; and in orange, the inverse problem model was fed with the regressions developed by Wells and Coppersmith (1994). Dashed vertical lines indicate the averaged values for each approach. (**a**) Results for the half width, *b*, (**b**) Results for the uplift speed, $W_0$, (**c**) results for the half length, *L* and (**d**) results for the half duration, *T*.

Two approaches were taken to generate an input for the inverse problem model and the results were compared, see Fig. 5. In the first approach, the magnitude of the event was not taken into account and the inverse problem model was fed with the total observed range of values for each effective earthquake property by Wells and Coppersmith^20^. Thus, for each property the range spans from the minimum to the maximum observed values, half effective length of the fault $L=[1-400]$ km; half width $b=[1-90]$ km; maximum surface displacement $d=[0.05-10]$ m; half duration $T=[0.1-25]$ s^28,29^.

The length of the effective displacement 2*L* was estimated from Fig. 9 in Wells and Coppersmith^20^, the area from figure 16^20^, allowing the further calculation of the width 2*b*. The maximum vertical displacement was estimated from Fig. 11 in Wells and Coppersmith^20^. Note that the predictions for the characteristics of the earthquake are based on empirical values, which may include significant scatter^20^. Wells and Coppersmith^20^ regressions were made based on a dataset composed of more than 400 earthquakes with magnitudes ranging from 5.2 to 8.1.

The second approach is based on the regressions developed by Wells and Coppersmith^20^ that relate moment magnitude (Mw) with effective properties of the earthquakes by:

3 $$\begin{aligned} \begin{aligned} \log {SRL}= c_1+c_2 Mw, \quad \quad c_1=-3.22,\quad c_2=0.69\\ \log {RA}= c_3+c_4 Mw, \quad \quad c_3=-3.49,\quad c_4=0.91\\ \log {MD}= c_5+c_6 Mw, \quad \quad c_5=-5.46,\quad c_6=0.82 \end{aligned} \end{aligned}$$

where *SRL* stands for surface rupture length, *RA* for rupture area (Table 2A Wells and Coppersmith^20^), and *MD* for maximum displacement (Table 2B Wells and Coppersmith^20^).

The applied parameterizations provided a single value for each effective fault property, which was divided by 2 and multiplied by 2.5 to define the limits for the input ranges of the inverse model. These coefficient values are set after visual inspection of the information published by Wells and Coppersmith^20^ in order to simulate the scatter found in the data^3^.

In the spectrogram associated with the pressure signal, we identified maximum and minimum limits for the potential values of the frequency distribution of the first acoustic mode at 0.8 and 10 Hz respectively. With this information, the algorithm identified four potential first mode frequency distributions that lie within the established frequency range. Then, the relevant part of the acoustic disturbance was selected by inspection of the short-time energy distribution. Finally, the model was run producing a set of 40 solutions (a set of 10 solutions was calculated for each potential orientation of the fault) for each property of the effective ground uplift associated with the tectonic event: *L*, *b*, *T* and uplift speed $W_0$, see Fig. 5.

The regressions found in Wells and Coppersmith^20^ were applied to the estimated magnitude of the event (Mw) leading to: $SRL=25.29$ km, RA = 404.6 km^2^, width $b=15.99$ km and $MD=1.08$ m. The retrieved properties by the inverse problem model fed with the total observed range of values in Wells and Coppersmith^20^ for the effective properties (where the magnitude of the event was not considered as input) are $L=92.43$ km, $b=12.28$ km, $T=7.28$ s and $d=1.03$ m ($2TW_0$). For the case where the model is fed with property ranges based on the regressions found in Wells and Coppersmith^20^, the retrieved values are: $L=17.59$ km, $b=6.78$ km, $T=8.59$ s and $d=1.25$ m, see Fig. 5. Both approaches for the input of the model led to sets of results that lie in the same order of magnitude. The calculated effective half length of the fault, in the case of the magnitude of the event not taken into account as input, is significantly larger than for the case where the magnitude is used as input. For the remaining four introduced earthquakes, only the ‘1st’ and ‘2nd’ sets of features are used along with RFR and SVR, since they led to the best regression accuracy results, and the obtained magnitude results averaged to provide a single final value. The ‘2nd’ and ‘3rd’ sets of features led to the highest classification accuracy. Thus, they are applied and the resulting classifications compared, see Tables 5 and 6. Note that only the binary classification result is used as input for the inverse problem model. The average absolute binary classification error and the absolute mean regression error are 0% and 2.382 %, respectively.

**Table 5:** Results delivered by the ML algorithms.

Earthquake	Mw	Retrieved Mw	gCMT	Multi-class	Binary	h (m)	Distance (km)
16/02/2015	6.700	6.727	Thrust	(T) 100%	(V) 100%	4000	3200
21/12/2010	7.400	7.072	Normal	(N) 100%	(V) 100%	4500	2500
25/10/2013	7.100	7.017	Normal	(N) 100%	(V) 100%	5000	2900
14/03/2012	6.900	6.740	Normal	(T) 50% - (N) 50%	(V) 100%	5000	3150
29/09/2009	8.100	7.810	Normal	(N) 75%	(V) 100%	4000	4450

Distance refers to the hydrophone-epicentre separation, (V) stands for vertical earthquake, (H) for horizontal, (N) for normal and (T) for thrust. gCMT are the reported slip types. *h* is the average ocean depth between the epicentre and hydrophone.

**Table 6:** Properties retrieved by the inverse problem model: number of runs (sets of solutions, 10 solutions each set) carried by the model and the average computational time taken by each of the runs.

Earthquake	L (m)	b (m)	T (s)	$W_0$ (m/s)	Time/run (s)	Runs (#)
21/12/2010	26,500	9600	17.71	0.035	4.85	4
25/10/2013	26,400	9400	27.7	0.02	2.3	3
14/03/2012	14,800	7200	23.5	0.013	2.6	3
29/09/2009	93,100	11,300	12.73	0.135	2.92	5

#### Synthetic signal analysis

In this section, we report on the performance of the application of classification algorithms on synthetic pressure signals generated by Eq. (4) found in Mei and Kadri (2018)^1^. The applied ML algorithms were trained with real earthquake acoustic signals. The bottom pressure signals induced by the acoustic waves in the far field are described by

4 $$\begin{aligned} p = \rho W_0 |A| \dfrac{2^{7/2} c }{\sqrt{\pi ^3 x_0 k}} \sin {\left( k b\right) } \sin {\left( {{\hat{\Omega }}} T\right) }, \end{aligned}$$

where $\rho$ is the water density, *A* is the two dimensional envelope^1^, *k* is the wave number and ${\hat{\Omega }}$ is the frequency. Note that only the pressure induced by the first acoustic mode is taken into account, as it carries most of the energy and information about the source^1^. The analytical solution used to generate the synthetic signals takes simplifications such as constant speed of sound or rigid and flat seabed which are discussed in other studies^1,3^.

The slender fault properties and relative coordinates used to calculate the pressure signals by Eq. (4) were randomly generated within the ranges observed by Wells and Coppersmith (1994)^20^: water column depth $h=[3000-4000]$ m; total distance from the centre of the fault to the virtual hydrophone $r=[1000-4000]$ km; $L=[10-200]$ km; $b=[2-(L/8)]$ km; $d=[0.03-10]$ m; $T=[2-20]$ s. Here, 20 synthetic signals were generated and windows of 300 seconds extracted after the arrival of each pressure disturbance to the virtual hydrophone. Then, the four types of signal vectorization considered in this study were performed and binary classification was applied with 5-fold hyperparameter grid search. As a result, the RFC and SVM algorithms identified the synthetic signals as incoming from vertical motion earthquakes (‘1’) with an accuracy of 100%, as expected.

We studied the effects of adding and shuffling different numbers of synthetic signals into the dataset (composed only by real earthquake recordings) used to train the classification algorithms, for more details see ‘Machine learning application on synthetic signals analysis’ subsection in ‘Sensitivity analysis’ section in the ‘Supplementary materials’. The overall accuracy of the classification algorithms increased due to the addition of synthetic signals to the training dataset. However, when the amount of added synthetic signals became $\approx 10\%$ (20 synthetic signals in the studied case) of the dataset size, a trend of increased bias was noticed, leading to a higher incorrect classification of the horizontal slip events (‘0’). Thus, it is recommended to include a number of synthetic signals smaller than 10% of the total dataset size in order to optimise the classification accuracy for both tectonic event slip types.

## Discussion

We applied a set of techniques capable of analysing acoustic pressure signals induced by underwater earthquakes and calculated the effective fault size and dynamics in almost real-time. To fulfill this goal, we studied a dataset composed of 201 earthquake signals recorded by the IMS hydro-acoustic network. Furthermore, we compared four different methodologies to extract relevant features from acoustic signals incoming from submarine earthquakes, based on statistical moments, time series analysis, power spectrum analysis, wavelet transform coefficients analysis and cepstral coefficients were considered and compared. Along with the vectorization methodologies, we applied two classification ML algorithms (RFC and SVM), which were able to discriminate vertical motion events with over 70% classification accuracy. Amongst the tested methodologies, the wavelet transform feature extraction technique in combination with SVM led to the highest classification results accuracy for both binary and multi-class scenarios.

Regarding the three-type classification, included as a secondary result, there is a low classification of ‘normal’ events, which can be caused by an unbalanced dataset^31^, where the balance between ‘horizontal’ and ‘vertical’ events was prioritised, leaving around half of the set to be split into the two new classes (thrust and normal). The inclusion of more signals induced by ‘normal’ earthquakes or the use of penalised ML algorithms could yield higher accuracy results. Furthermore, the application of feature selection algorithms instead of using feature sets based on previous studies has the potential of improving the ML algorithms accuracy.

Additionally, regression ML algorithms were applied to the vectorized signals dataset to estimate the magnitudes of the associated tectonic events. The ML algorithms delivered better predictions than the mean value of the dataset, which was confirmed by the SSE values. It is remarkable that the precomputed vectorized dataset along with the ML algorithms take less than one second on a standard desktop machine to deliver the source magnitude and slip type estimations. Finally, the magnitude and slip type retrieved by the ML algorithms can be used to feed an inverse problem model to perform real-time calculations of the fault effective size and dynamics. In this study, the depth dependence of the classification accuracy has not been analysed (only shallow earthquakes have been selected to reduce uncertainties) which is intended to be done in the future in order to expand the studied dataset and potentially improve the accuracy of the model.

The size of the dataset is a limitation. Only earthquakes that meet specific conditions are considered, reducing significantly the cases that serve our purpose. Thus, this study is presented as a proof of concept to show the applicability of a combination between ML algorithms and semi-analytical solutions to infer the properties of submarine tectonic events from acoustic radiation. Another issue that we would like to study in the future is the relations between the magnitude prediction results and the type of studied earthquakes, where possible patterns might shed light on the process understanding.

## Methods

### Data and instrumentation

#### Instrumentation

We used data from three hydrophone stations deployed by CTBTO: HA01 (Cape Leeuwin), HA08 (Diego Garcia) and HA11 (Wake Island). Each station consists of two triplets except HA01 which has a single triplet^27^.

**Figure 6:**
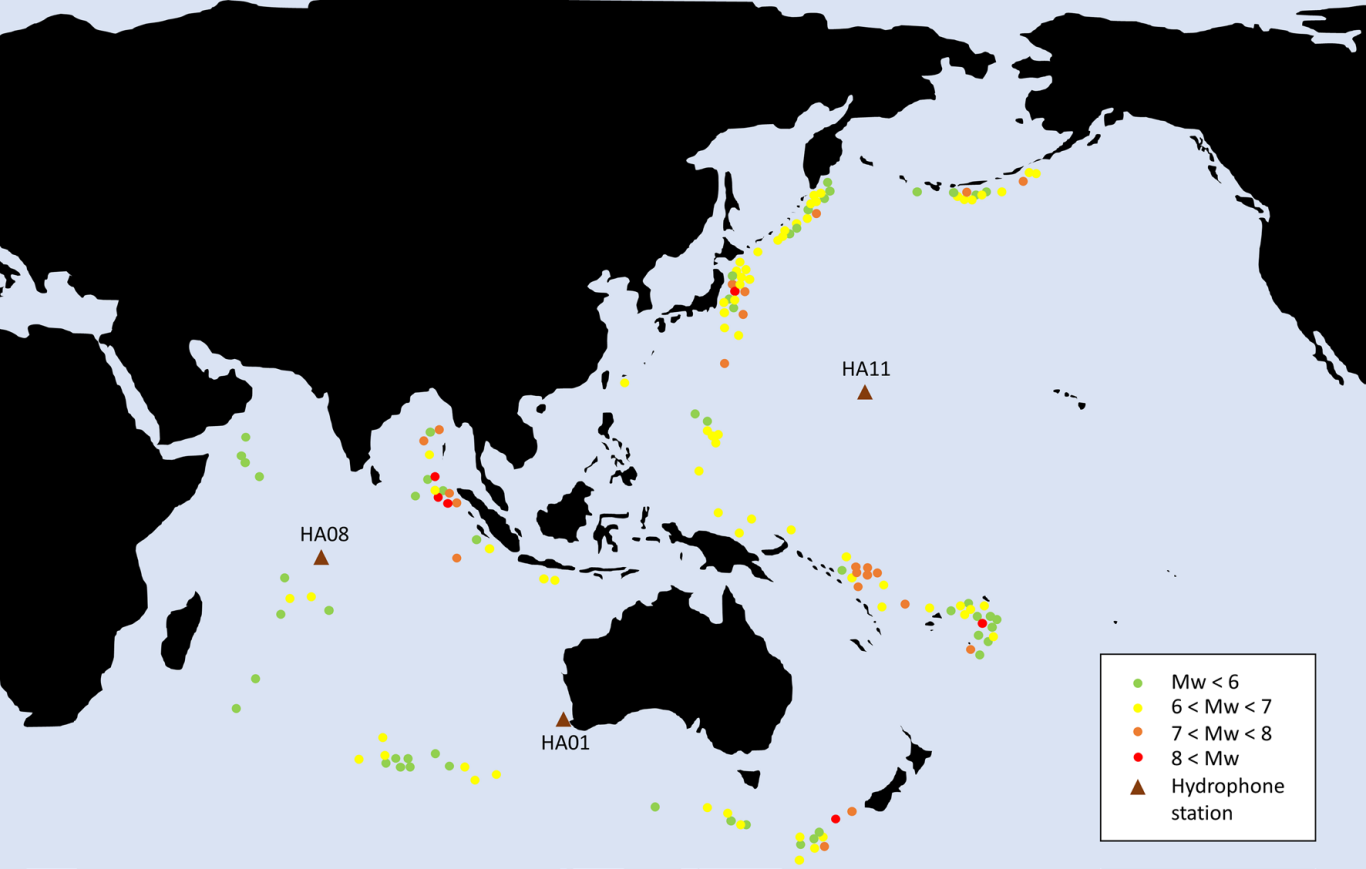
Geographic distribution of the earthquake epicentres associated with the signals in the studied dataset. Image generated in Microsoft PowerPoint 2016.

The hydrophones are suspended at a depth corresponding to the SOFAR channel axis and anchored to the seabed via a riser cable, which is kept under tension by a sub-surface buoy^32^. The recordings used in this study are extracted from the International Monitoring System (IMS) database, which are originally recorded by the IMS *‘H11N1’*, *‘H11S1’*, *‘H01W1’*, *‘H08S1’* and *‘H08N1’* hydrophones, see Fig. 6. The calibration files for the mentioned instruments, provided by CTBTO, show a steep roll-off below 0.1 Hz in the instrument response curve, and consequently, in this study, the analysis of frequency bands below 0.1 Hz is not considered. The data has a sampling frequency of 250 Hz.

#### Dataset

Long distances from hypocenter to epicentre can induce distortion and attenuation on seismic waves leading to higher uncertainties in measurements. In order to minimise these effects, we considered only acoustic signals associated with shallow earthquakes. Different values for the maximum hypocenter depth below the seabed are used to define shallow earthquakes in the literature, such as 60 km^5,33^ or 100 km^7^. We only included earthquakes with hypocenter located at less than 60 km deep under the sea bed in the dataset. The dataset was built with 201 acoustic recordings induced by ‘shallow’ tectonic events, listed in the ‘List of earthquakes’ section in the ‘Supplementary materials’. The data was provided by CTBTO.

To minimise diffraction effects only earthquakes with shortest transects, that do not cross through lands, were analysed, see Fig. 6. In addition to the previously mentioned constraints, only underwater earthquakes are studied, which significantly narrows down the search.

The earthquake’s source types of slip and magnitudes were labelled based on data reported by gCMT^28,29^. The slip type labelling of the dataset depends on the slip angles of the fault planes. For slip angles 0° and 180° the faults were classified as pure strike-slip, whereas for 90° and − 90°, faults were classified as pure dip-reverse and pure dip-slip normal, respectively (± 20°).

### Digital signal processing methods

#### Signal vectorization (feature extraction)

Signal vectorization is the process of reducing the size of the acoustic data set by determining category variables that define the sound type or identity of the sound source. It is important to decrease the signal dimensionality, as the training dataset grows exponentially as a function of the number of variables in the feature vector^8^.

Previous studies on ML approached the classification of CTBTO acoustic signals by operating on features automatically extracted by the organization^10,25^. However, the mentioned studies had to handle missing values for some features^9^. To overcome this difficulty, raw acoustic data were analysed and a signal processing algorithm, for signal vectorization was developed, ensuring that there were no missing values in the feature vectors. It has been reported that a single feature cannot train the classifiers efficiently^8^. Thus, we considered and compared five types of features: temporal (obtained directly from the time series)^8–10^, spectral (obtained from the power spectrum)^8–10^, cepstral^8–10,12,34^, statistical (statistical moments applied to the times series)^9,10^ and wavelet transform type^35^.

Inspired by previous studies^8–10,35^, four different sets of features were built and tested along with the considered ML algorithms. The first and second sets consist of a combination of four types of features (temporal, spectral, statistical and cepstral); the third set is composed only by wavelet transform extracted features; and the fourth set consists of cepstral features only, as shown in Table 7.

**Table 7:** Studied feature sets, tested on the classification and regression algorithms.

Feature set	Feature type				
	Temporal	Statistic	Spectral	Cepstral	Wavelet
1	Max. amplitude, total short-time energy, zero- crossing rate	Kurtosis, skewness		Maximum, variance	
2	Max. amplitude, total energy, zero-crossing rate	Kurtosis, skewness, std. deviation	Power spectral density (PSD) mean, PSD coefficient, skewness, roll-off, PSD std. deviation, PSD skewness	Variance, min kurtosis, max. kurtosis, spectral coefficient, median	
3					Std. deviation, average short-time energy, power ratio, zero-crossing rate
4				Variance, min. coefficient, skewness, kurtosis, max. coefficient, median	

#### Signal identification

We estimated the signal travel times from source to receiver by dividing the distance between the earthquake epicentre and the recording instrument by the speed of sound, considered constant ($c=1500 m/s$). The centroid times and location coordinates were obtained from the Harvard global CMT database.

For each recording (*x*(*t*)), the part of the signal carrying most of the information was identified by finding the point with maximum absolute amplitude (|*x*(*t*)|) and extracting a window composed of *N* samples at each side of the selected point. Short-time energy analysis was performed to define the potential extracted signal lengths (2*N*), for more details see ‘K-fold and grid search’ subsection in ‘Sensitivity analysis’ section in the ‘Supplementary materials’. The expression for the short time energy ($E_f$)^36^ has the form

5 $$\begin{aligned} E_f = \sum _{n=0}^{N_e-1} x^2(n) \end{aligned}$$

where $n=0$ and $(N_e-1)$ represent the limits of each frame, $N_e-1$ is the length in samples. The time-frames were chosen to be five seconds long ($N_e=1250$ samples), which was chosen to be shorter than the shortest earthquake duration in the studied dataset.

We set $N=20,000$ samples (160 s) as the maximum considered half window length for feature extraction. This choice was made after observing that it exceeds the duration of most signals in the dataset. Several values of *N* were tested, ranging from 2500 to 20,000 samples, for more details see the ‘K-fold and grid search’ subsection in the ‘Sensitivity analysis’ section in the ‘Supplementary materials’. The minimum tested half window length was 2500 samples, since it was intended to only analyse the frequency behaviour of the signals down to 0.1 Hz.

#### Frequency behaviour analysis and time series features

**Figure 7:**
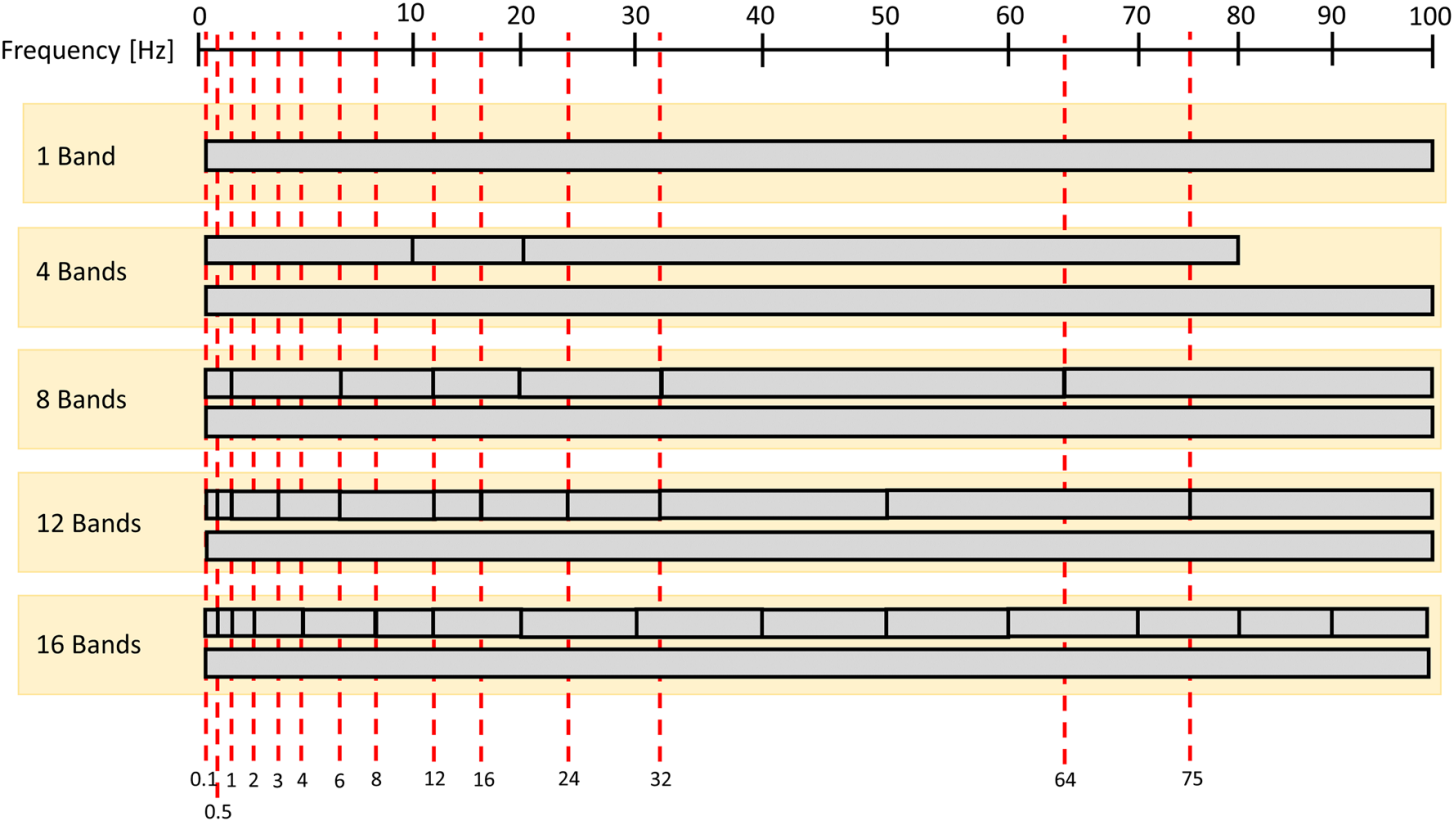
Considered frequency spectrum subdivisions.

In order to decompose the signals in different frequency bands and study their behaviour at different points of the frequency spectrum, we applied Butterworth band-pass filters, which have been reported to have good performance for classification purposes^37^. Then, the extracted features are statistical moments applied to the filtered time series such as standard deviation, kurtosis, skewness, maximum amplitude and zero-crossing rate. Additionally, the short-time energy distribution was computed for each band, with a five seconds window width, and the maximum and total short-time energy were calculated. Several potential divisions of the frequency spectrum (see Fig. 7) were analysed, tested and compared, for more details see the ‘K-fold and grid search’ subsection in the ‘Sensitivity analysis’ section in the ‘Supplementary materials’.

Based on the fact that earthquakes with vertical motion components excite lower frequencies due to the compression of the water^1^, we performed more subdivisions at lower frequencies than at higher frequencies. Moreover, slow events show lower frequency excitation, in the range of 0.1 to 10 Hz^7^. Some of the tested frequency subdivision approaches taken in this study were inspired by previously published studies^10,14^, see Fig. 7.

We analysed the introduced subdivisions of the relevant part of the frequency spectrum and the optimal window size (2*N*) by running the classification models SVM and RFC on the 2-type class labelled dataset. In this analysis, only the set of features ‘1’ was used, along with every combination between the considered window sizes (2*N*) and spectrum division approaches. It can be seen that the standard deviations lie below 15% for SVM and 12% for RFC on the tested cases. In addition, the variations on accuracy along with the different considered scenarios are lower than 3%. Finally, we selected 15000 samples as the half window size (*N*) and the eight frequency band spectrum division approach for the final set-up. This choice was made based on the results of the carried sensitivity analysis, for more details see the ‘K-fold and grid search’ subsection in the ‘Sensitivity analysis’ section in the ‘Supplementary materials’.

#### Spectral and cepstral features extraction

The signals were further processed to extract spectral and cepstral features. The cepstrum can be calculated by taking the inverse Fourier transform after applying a natural logarithm to the Fourier transform of a signal. The logarithm maps convolution in the time domain to addition in the frequency domain.

While for speech the optimum window length for framing is [16–32] ms, for acoustic sounds in the ocean, the window length may be different. Thus, the signal was sliced into frames of ten seconds duration (in order to be able to capture frequencies down to 0.1 Hz). We set 50% overlap^12,34^ amongst the frames and Hamming window was applied^38^. Then, Fourier transform was computed and the power spectrum for each frame was calculated by

6 $$\begin{aligned} P =\dfrac{conj(X(s))X(s)}{NFFT}, \end{aligned}$$

where *NFFT* is the transform length in samples of the signal used by the fast Fourier transform, which in this case is 4096 in order to provide enough frequency resolution. Because of the nature of the FFT algorithm, *NFFT* was chosen to be multiple of two to make the algorithm more efficient.

The next step was to compute filter banks using triangular filters, which were applied on a scale inspired by the Mel-scale to the power spectrum in order to extract frequency bands. The Mel-scale aims at mimicking the nonlinear human ear perception of sound. Cepstral coefficients were used in the literature for the classification of sound sources in underwater environments due to their robustness to noise^8–10,34^. Note that the Mel scale was designed for higher frequencies than the bands we are interested in, i.e. 0.1–10 Hz. Therefore, we modified the constants in the conversion formula to have an almost linear mapping from Hertz (*f*) to Mel frequency (*m*) in the range of 0.1 to 10 Hz and logarithmic mapping over 10 Hz, see Eq. (7). The conversion between Hertz and Mel frequency is done by

7 $$\begin{aligned} m =25.95\log {\left( 1+\dfrac{f}{7}\right) } \end{aligned}$$

The new constants are obtained from

8 $$\begin{aligned} C =\dfrac{f}{\log {\left( 1+\dfrac{f}{f_0}\right) }} \end{aligned}$$

where $f_0$ was chosen to be 7 Hz and $f=10$ Hz, thus, below 10 Hz the relation between Mel frequency and frequency in Hz is almost linear.

The applied triangular filters are highly correlated due to the overlap, and thus, we used the discrete cosine transform (DCT) to decorrelate the filter coefficients^12^. It is shown in the ‘Cepstral coefficients analysis’ subsection in the ‘Sensitivity analysis’ section in the ‘Supplementary materials’ that 12 cepstral coefficients capture most of the information carried by the signal. Finally, statistical moments (mean, maximum, kurtosis, skewness and variance) were applied to each coefficient band to reduce the number of features. The spectral features, listed in Fig. 7, were extracted from the one-sided power spectrum computed on the calibrated original signals.

#### Wavelet transform parameters analysis

The power distribution of the signals along with different frequency bands can be analysed by applying the discrete wavelet transform (DWT)^39^, of the form

9 $$\begin{aligned} W(a,b) = \dfrac{1}{\sqrt{|a|}}\int _{\infty }^{-\infty }x(t)\psi \left( \dfrac{t-b}{a}\right) dt, \end{aligned}$$

where *a* is the scaling parameter, *b* is the translational parameter and $\psi$ is the mother wavelet. Here, a given signal is projected onto a space defined by a set wavelets, which are function of frequency and time^40^,

Several studies approached the extraction of features by wavelet transform algorithms for different purposes, such as earthquake magnitude prediction by seismic waves analysis^18,41^ or source type classification of acoustic signals^24,35,39,42^. Even though numerous wavelet bases exist, we tested and compared only two discrete wavelets, Daubechies and Symlet, being two of the most popular wavelets in signal processing. Furthermore, the order of the wavelets was also analysed, which indicates the number of vanishing moments and is related to the approximation order and smoothness of the wavelet. After applying DWT, *n* sets of detail coefficients and one set of approximation coefficients ($n+1$ times each feature) were produced. Note that $n=6$ levels, has been used in previous studies^35^. In order to analyse the sensitivity of the ML algorithms to variations in DWT parameters, we tested a different number of levels [4–8] and wavelet orders [2–8], see ‘Sensitivity analysis’ in ‘Supplementary materials’. We found that the accuracy amongst the considered scenarios has a deviation of less than 5%. Nevertheless, we decided to use Symlet wavelet of order eight with six levels of coefficients as the final setup, since it provides a good balance between computational efficiency and accuracy.

Finally, after applying DWT, statistical moments were calculated for each extracted coefficient band or level ($n+1$): standard deviation, average short-time energy, power ratio between the first coefficient band and every other band and zero-crossing rate^42^, see Table. 7.

### Machine learning (ML) algorithms

In essence, ML algorithms learn from data using probability theory and can be grouped into two main categories: supervised learning (labelled dataset) and unsupervised learning (unlabelled dataset). In this study, we applied supervised learning, which can be further subdivided into classification and regression algorithms based on whether the target outputs are categorical (classification for slip type) or quantitative (regression for magnitude)^43^. In particular, two ML algorithms were explored, SVM and RFC^8–10,12^.

The first algorithm, SVM, is a classification technique that can additionally be used for regression purposes^44^. It uses a convex cost function, always reaching the global minimum of the cost function^12^. It is able to perform reliable classifications even with small datasets^45^. SVMs differentiate classes by finding the hyper-plane that splits them and maximize the margin between the closest point of each class and the hyper-planes. Each signal is vectorized and described by a point in a *n* dimensional space and a cost function is fully specified by the subset of training examples called support vectors. The output of the SVM is a set of weights, which in combination will predict the value of the outcome. Because of the non-linear nature of the studied process, a non-linear kernel has been selected, ‘Radial Basis Function’ (RBF), being this a type of Gaussian kernel, that has been proven to provide good accuracy and efficiency^9^. SVM are commonly found in the literature to classify acoustic signals in the ocean^8–11,11,12,44,46^.

The second studied algorithm, RFC (introduced in 1995)^47^, is a technique based on decision trees that operate as an ensemble. It can perform classification and regression^48^ by splitting a dataset into smaller data subsets, while an associated decision tree is incrementally developed. The final result is a tree with decision nodes and leaf nodes. Decision nodes have two or more branches and leaf nodes represent a classification or decision. Each individual tree in the random forest delivers a class prediction and the class with the most votes becomes the model’s prediction.

It is important to remark that, regularization (normalization) of the features was carried before the application of SVM, due to significant differences in the order of magnitude between the feature values. However, it is not necessary for the application of RFC. In addition, we used validation k-fold technique^12^ (10-fold) in combination with grid search (5-fold) to identify the best model hyper-parameters and test the accuracy of each algorithm, for more details see ‘k-fold and grid search’ subsection in ‘Sensitivity analysis’ section in the ‘Supplementary materials’. Finally, the accuracy of all fold tests was averaged and precision and standard deviation results were calculated.

### Inverse problem model

A semi-analytical inverse approach is employed assuming the fault is single (A similar solution can be derived for multi-fault rupture based on Williams et al. 2021^49^), slender and uniform, and the seabed is flat, which allows the calculation of the effective fault characteristics in almost real-time from the pressure acoustic signature. The three-dimensional wave equation governs the propagation of acoustic waves in slightly compressible fluids^50^:

10 $$\begin{aligned} \dfrac{\partial ^2\phi }{\partial x^2} + \dfrac{\partial ^2\phi }{\partial y^2} + \dfrac{\partial ^2\phi }{\partial z^2} = \dfrac{1}{c^2} \dfrac{\partial ^2\phi }{\partial t^2}, \end{aligned}$$

where $\phi$ is the velocity potential, the velocity field is defined by $\mathbf {u} = \nabla \phi$. *c* is the speed of sound in water. With standard boundary conditions the problem of the propagation of acoustic waves induced by the vertical motion of a slender fault was constructed. At the surface, the pressure is assumed to be zero and uniform,

11 $$\begin{aligned} \partial \phi /\partial z = 0 \,\,\,\,\,\, at \,\,\,\,\,\, z=h, \end{aligned}$$

On the seabed ($z=0$), a piston model simulates the vertical displacement of the fault by

12 $$\begin{aligned} \frac{\partial \phi }{\partial z}={\left\{ \begin{array}{ll} W_{0}\tau (t) &{} |x|<b,\,|y|<L\\ 0 &{} \text {elsewhere} \end{array}\right. },\,\,\,\,\,\,\,\,\tau (t)={\left\{ \begin{array}{ll} 1 &{} -T<t<T\\ 0 &{} |t|>T \end{array}\right. }. \end{aligned}$$

The effective earthquake is assumed to have rectangular slender shape, with a length of 2*L* and width 2*b*, where $b/L\ll 1$ is the slenderness parameter.Due to the assumption of slender body, multiple scales theory can be applied and an analytical solution is reached [Eq. (6.1) in Mei and Kadri^1^].

The derived solution^1^ comprises a summation of a countable infinity of acoustic modes. However, for brevity, only the bottom pressure due to the leading acoustic mode is considered, which is the most energetic^50^. The solution provides a relation for the bottom pressure induced by the vertical motion of the slender fault in the far-field.

Note that, the slender fault geometry and dynamics considered here represent an effective vertical motion caused by the more complex earthquake rupture dynamics. The fault is assumed to move vertically upwards with a constant speed $W_0$, for a time duration 2*T*^1^. Thus, inferring the presence of vertical motion components in the studied tectonic events is of major importance for the application of the model.

An array with the potential combinations for $x_0$ and $y_0$ (orientation of the fault) can be defined considering that, the detected acoustic radiation induced by the earthquake can be triangulated by the hydrophone triplet to infer the distance to the source. The frequency distribution of the first acoustic mode is then calculated, which is a function of time *t* (relative to the eruption time $t_0$), depth *h*, and every potential orientation of fault ($x_0$ and $y_0$). Only the obtained frequency distributions that lie within the range defined by visual inspection of the spectrogram are considered and define the number of sets of solutions provided by the model.

**Figure 8:**
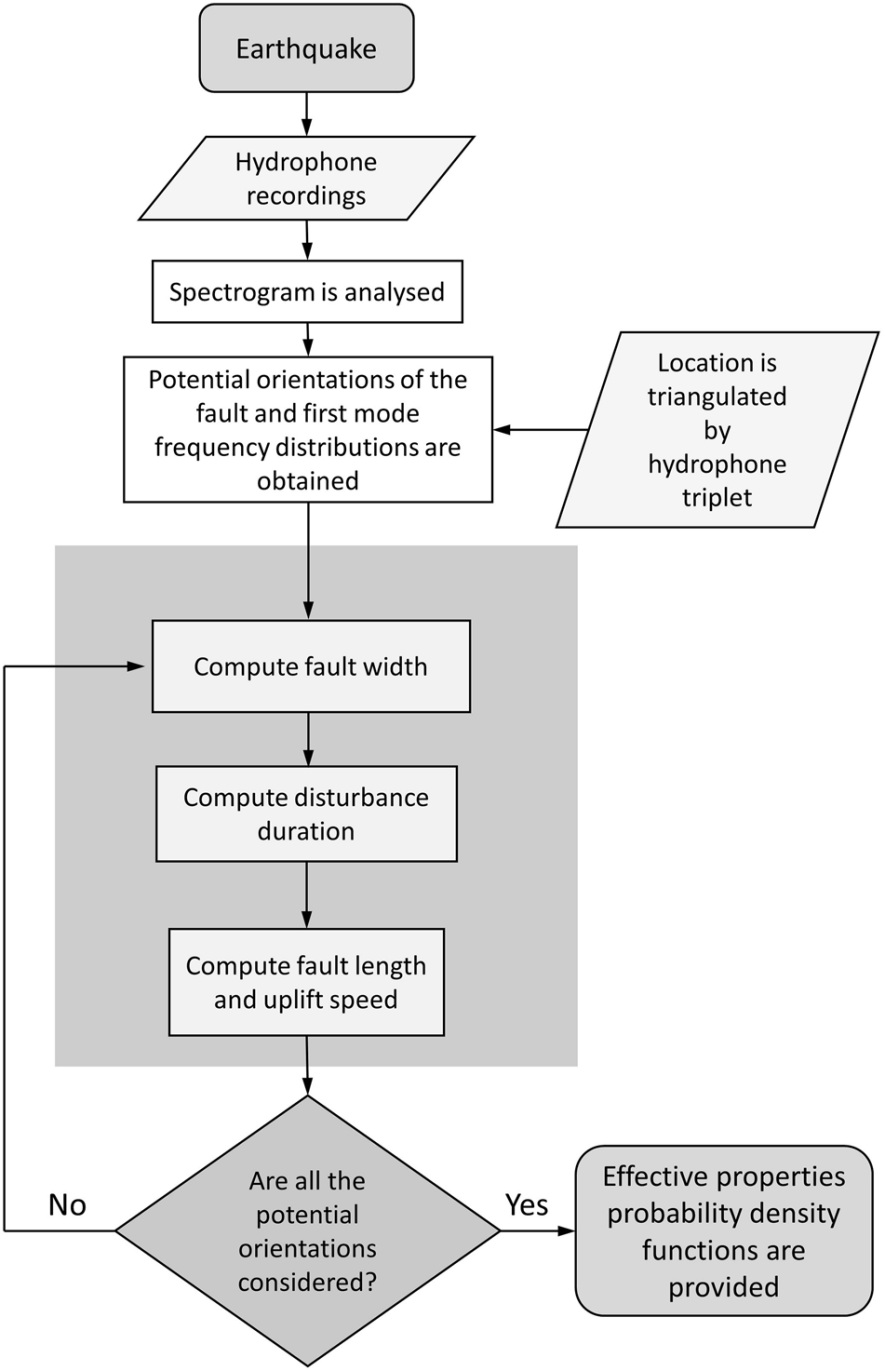
Inverse problem model process.

To identify the beginning and end of the disturbance in the recordings, the short-time energy distribution is analysed. Then, pressure points from the acoustic signal need to be chosen to retrieve the source properties^3^. Envelope tracking algorithms are applied since working with points close to the envelope reduces the associated uncertainties and the obtained pressure points are associated with the calculated potential first mode frequency distributions.

The magnitude estimated by the ML algorithms is used to generate the ranges that will feed the inverse problem model and confine the potential solutions for the effective geometry and dynamics of the tectonic event. Finally, the steps described in Gomez and Kadri^3^ section 3, are taken for each of the selected frequency distributions to retrieve *b*, *L*, *T* and $W_0$, see Fig. 8. Note that the calculation of ten solutions for each earthquake property and potential frequency distribution is a choice made to balance the computational effort and accuracy and can be modified. The solutions are plotted in four probability density functions along with the mean values, see Fig. 5. The fault geometry and dynamics are estimated within a few seconds on a standard desktop machine.

## Supplementary Information

Supplementary Information.
