# Supplementary material for: Earthquake source characterization by machine learning algorithms applied to acoustic signals
Source: Sci Rep. 2021 Nov 29;11:23062. doi: 10.1038/s41598-021-02483-w (PMC8630080; doi:10.1038/s41598-021-02483-w)
Supplement: Supplementary file 1 — Supplementary Information. [file 41598_2021_2483_MOESM1_ESM.pdf]

Supplementary Materials: Earthquake source characterization by machine learning algorithms applied to acoustic signals

Bernabe Gomez[1] **and Usama Kadri**[1,*]

[1]School of Mathematics, Cardiff University, Senghennydd road, Cardiff CF24 4AG, UK
[*]kadriu@cardiff.ac.uk

Multi-class classification

To further study the relations between earthquake slip types and their associated acoustic signals, the dataset was divided into the classes described in the manuscript. We tested both proposed classification algorithms along the four considered feature sets on the multi-class labelled dataset, where a 'One-versus-all' technique was applied, generating as many binary classifiers as label types and testing every class against the rest.

Features	SVM accuracy [%]	RFC accuracy [%]
**1**	58.19 ± 7.56	58.69 ± 7.82
**2**	59.67 ± 6.66	59.14 ± 7.89
**3**	63.67 ± 11.00	64.14 ± 8.99
**4**	59.69 ± 10.14	59.62 ± 9.54

Table 1. Accuracy and standard deviation [%] for multi-class classification using SVM and RFC.

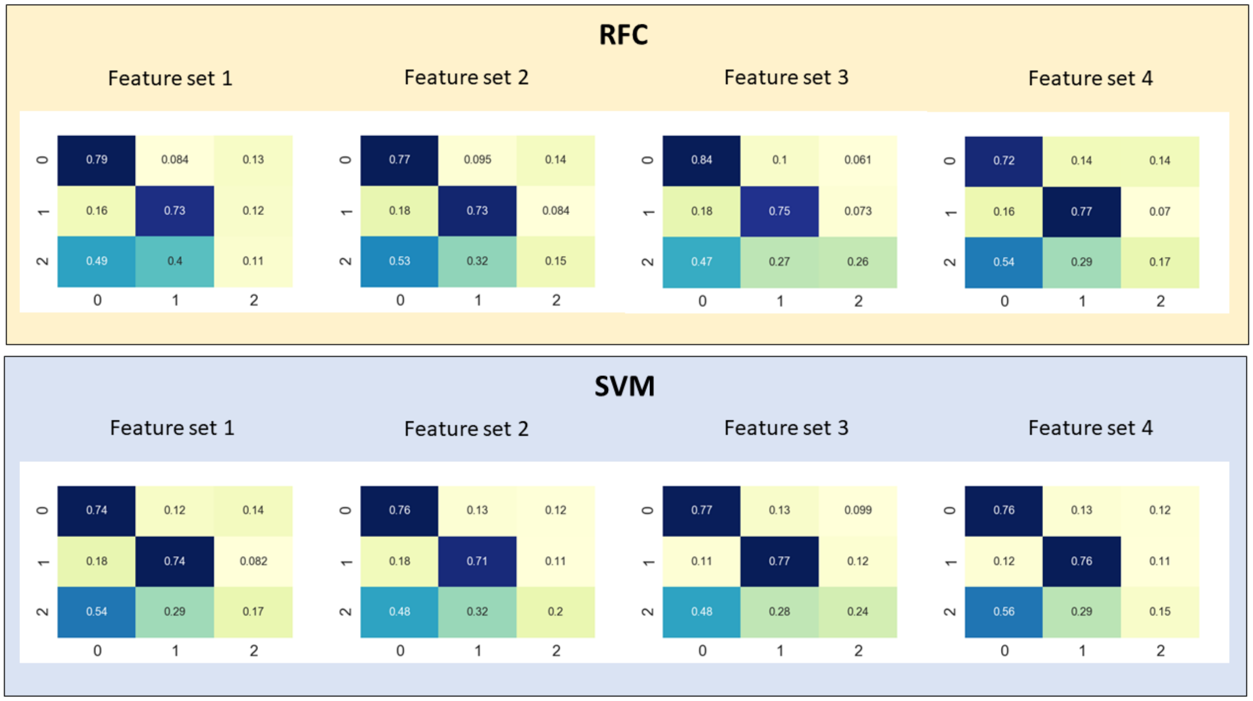

Figure 1. Multi-class classification confusion matrices for the tested feature sets and classification algorithms. ‘0’ stands for strike-slip events, ‘1’ for thrust events and ‘2’ for normal events.

In addition, the frequency of each moment magnitude per type of studied earthquake is plotted in Fig. 2.

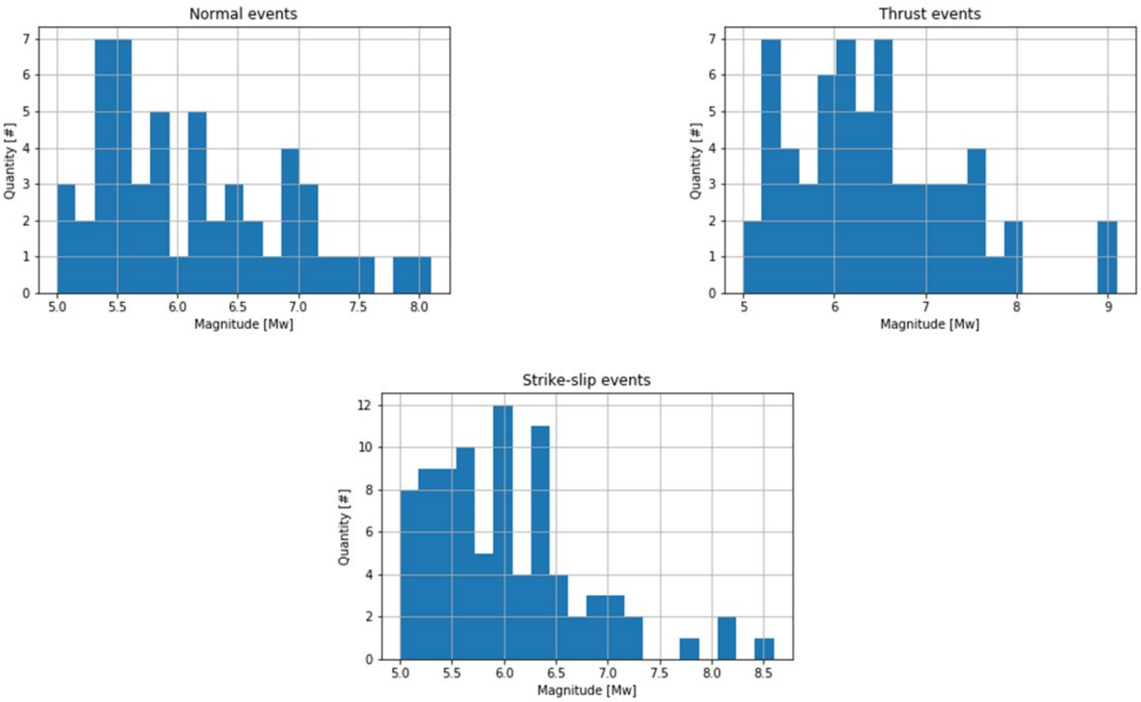

Figure 2. Dataset absolute moment magnitude frequency for each studied type of earthquake.

Sensitivity analysis

To understand the behaviour of the introduced classification algorithms under different input and setup conditions, we carried a sensitivity analysis.

K-fold and grid search

10-fold technique was applied for validation purposes on the classification and regression algorithms, see Fig. 3.

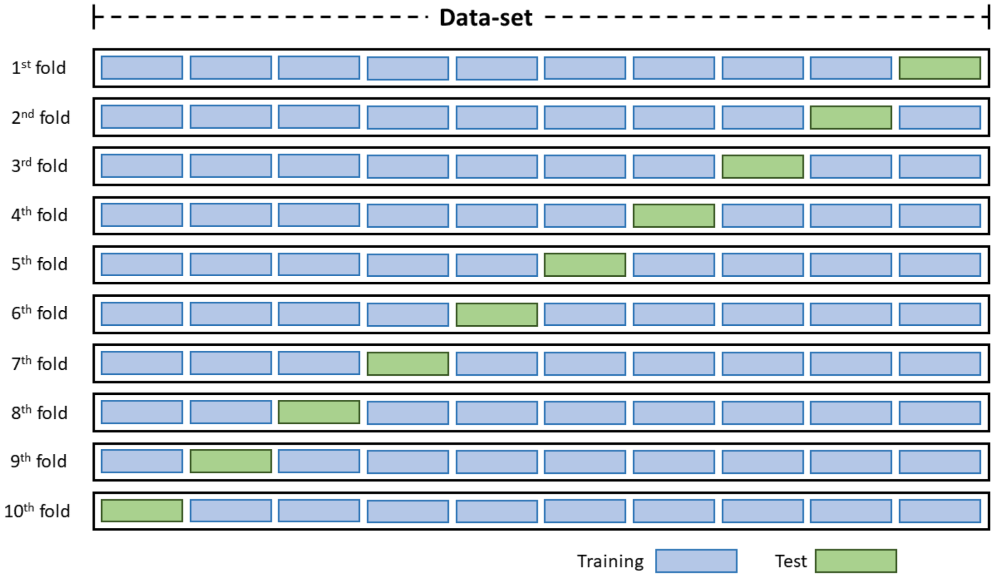

Figure 3. 10-fold scheme used for validation of the ML algorithms.

Additionally, for each validation fold, we applied 5-fold grid search on the training sets, see Fig. 4. This technique was used to identify the optimal hyper-parameter setup for the ML algorithms.

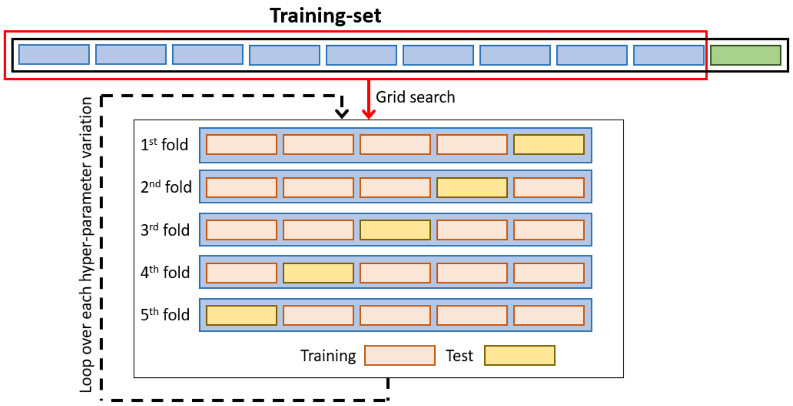

Figure 4. 5-fold grid search scheme.

For SVM and SVR, the iterated hyper-parameters in the 5-fold grid search are:

- **Kernel**: Radial basis function.
- **C (regularization parameter)**: 0.1, 0.2, 0.3, 0.4, 0.5, 0.6, 0.7, 0.8, 0.9, 1, 2, 10, 20.
- **Gamma**: 0.01, 0.015, 0.02, 0.025, 0.03, 0.04, 0.05, 0.1, 0.2, 0.3, 0.4, 0.5, 0.6, 0.7, 0.8, 0.9.

The regularization parameter represents the level of importance that is given to misclassifications in the algorithm training stage. The chosen kernel is 'RBF', which is the most generalized form of kernelization due to its similarity to the Gaussian distribution. The gamma parameter defines the reach of the influence of a single training example.

List of iterated hyper-parameters in grid search for RFC and RFR:

- **Number of estimators**: 10, 25, 50, 75, 100, 250, 500, 750, 1000.
- **Maximum depth**: 3, 4, 5, 6, 7.
- **Criterion**: 'gini' and 'entropy'.

The number of estimators refers to the number of trees in the forest. The maximum depth represents each tree depth, which is related to the number of splits done for the classification. The function to measure the quality of a split is the criterion, the considered criteria are 'gini' (Gini impurity) and 'entropy' (information gain).

Spectrum bands division analysis

The applied frequency limits for each considered decomposition of the spectrum are reported in Table 2.

**Band**	**1**	**2**	**3**	**4**	**5**	**6**	**7**	**8**	**9**	**10**	**11**	**12**	**13**	**14**	**15**	**16**
**Lower [Hz]**	0.1	0.5	1	2	4	8	12	20	30	40	50	60	70	80	90	0.1
**Upper [Hz]**	0.5	1	2	4	8	12	20	30	40	50	60	70	80	90	100	100
**Lower [Hz]**	0.1	0.5	1	3	6	12	16	24	32	50	75	0.1				
**Upper [Hz]**	0.5	1	3	6	12	16	24	32	50	75	100	100				
**Lower [Hz]**	0.1	1	6	12	20	32	64	0.1								
**Upper [Hz]**	1	6	12	20	32	64	100	100								
**Lower [Hz]**	0.1	10	20	0.1												
**Upper [Hz]**	10	20	80	100												
**lower [Hz]**	0.1															
**Upper [Hz]**	100															

Table 2. Tested frequency spectrum divisions.

For the sensitivity analysis, all considered divisions of the frequency spectrum were run along with the potential extraction window sizes for the case of binary classification and the accuracy results analysed, see Figs. 5, 6, 7, 8, 9, 10, 11, 12, 13, 14, 15 and 16.

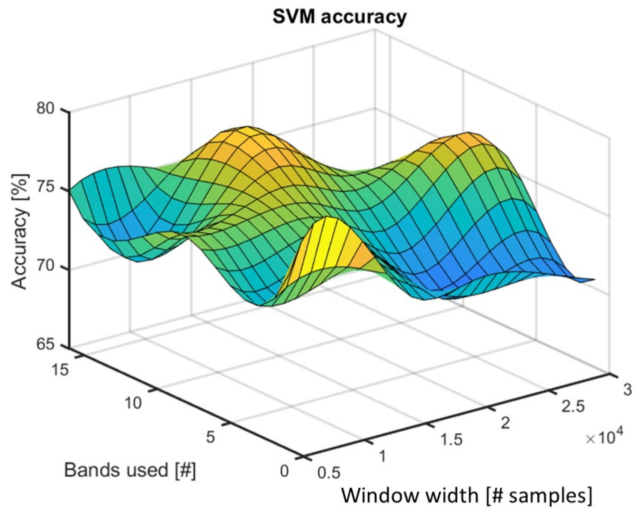

Figure 5. SVM accuracy [%] results for the potential combinations between different extracted window sizes and considered sets of frequency bands.

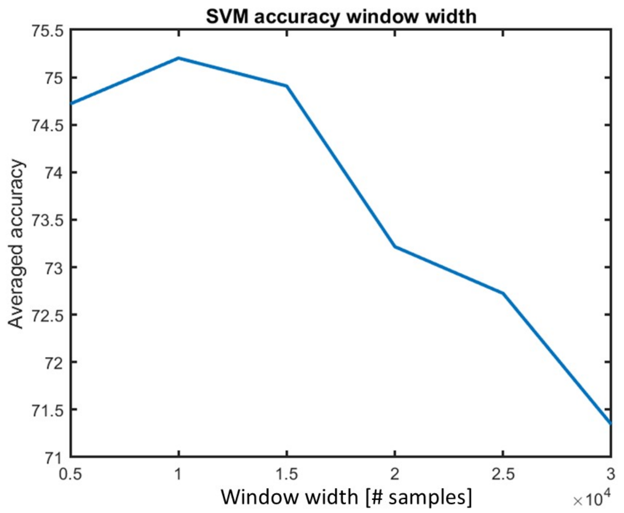

Figure 6. SVM accuracy [%] results averaged along with the considered spectrum divisions.

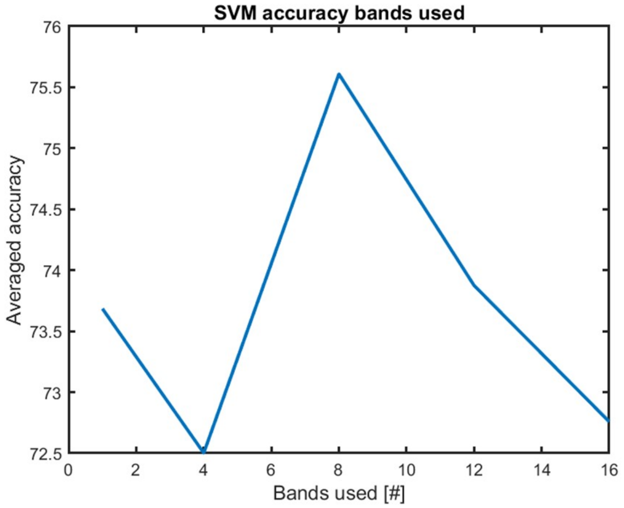

Figure 7. SVM accuracy [%] averaged results along with the considered extraction window sizes.

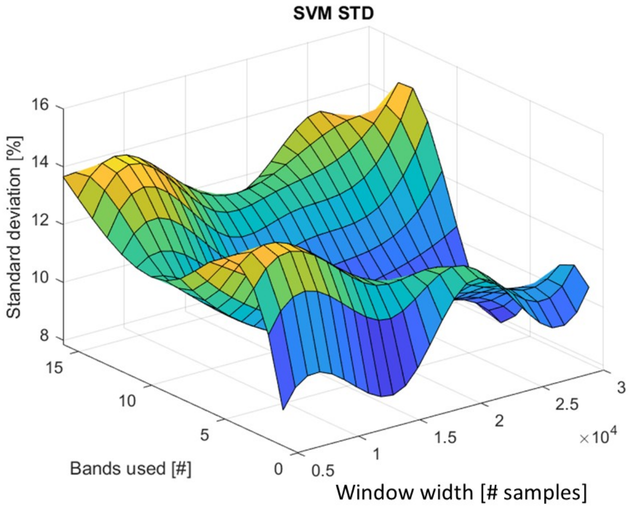

Figure 8. SVM standard deviation [%] results for the potential combinations between different extracted window sizes and considered sets of frequency bands.

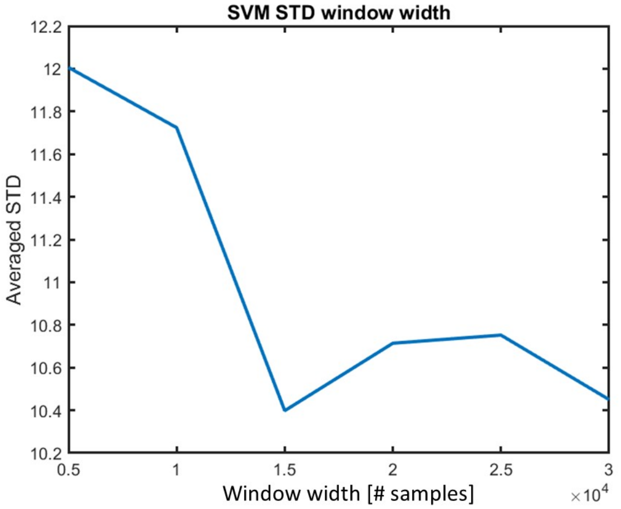

Figure 9. SVM standard deviation [%] results averaged along with the considered spectrum divisions.

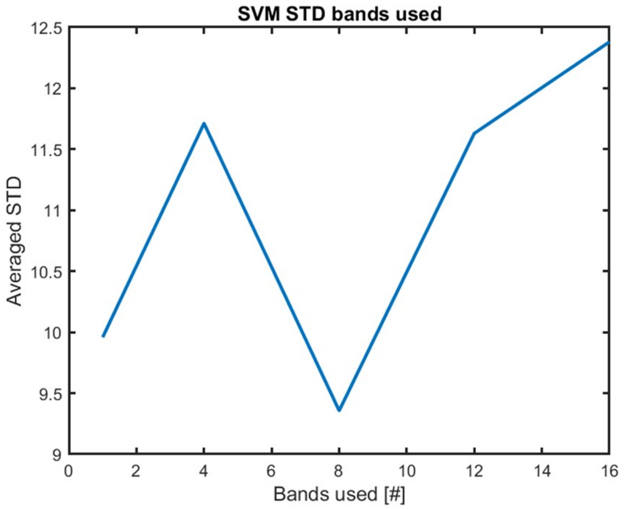

Figure 10. SVM standard deviation [%] averaged results along with the considered extraction window sizes.

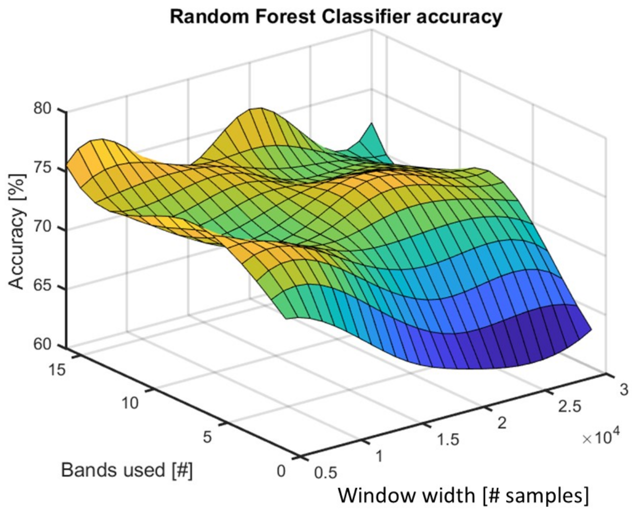

Figure 11. RFC accuracy [%] results for the potential combinations between different extracted window sizes and considered sets of frequency bands.

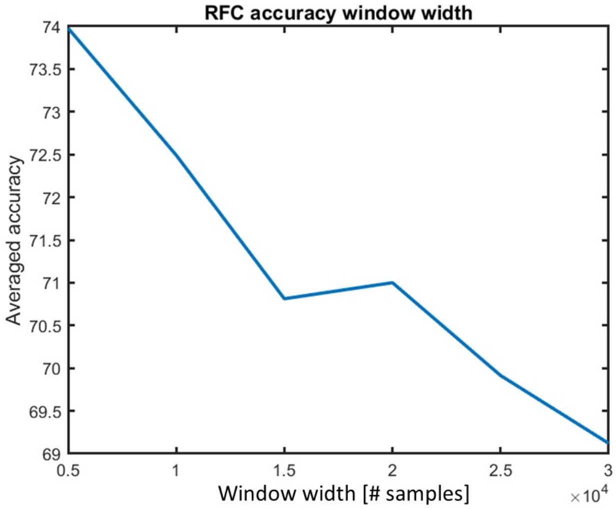

Figure 12. RFC accuracy [%] results averaged along with the considered spectrum divisions.

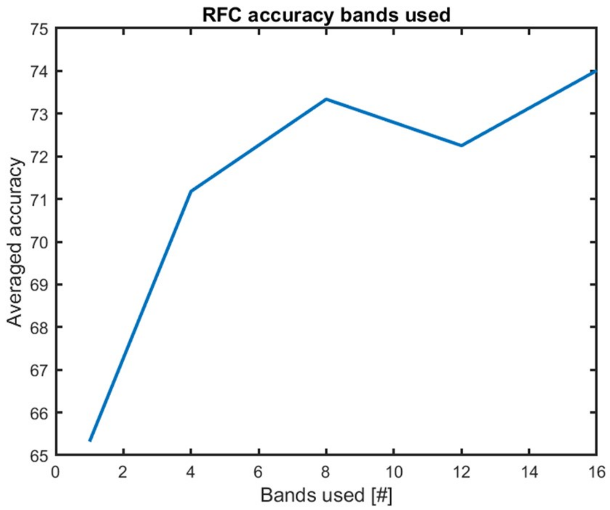

Figure 13. RFC accuracy [%] averaged results along with the considered extraction window sizes.

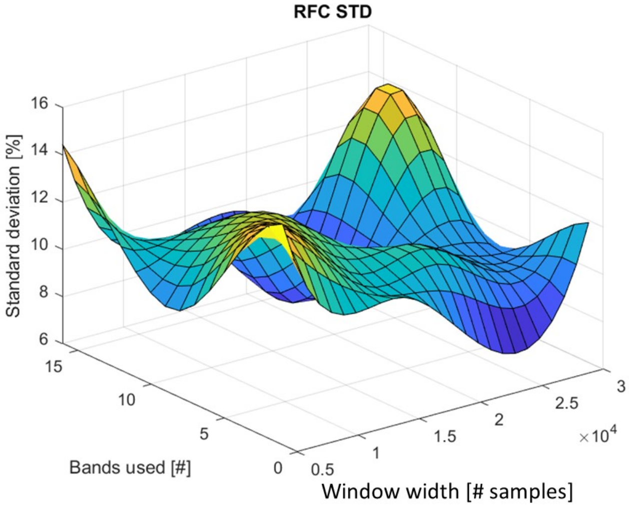

Figure 14. RFC standard deviation [%] results for the potential combinations between different extracted window sizes and considered sets of frequency bands.

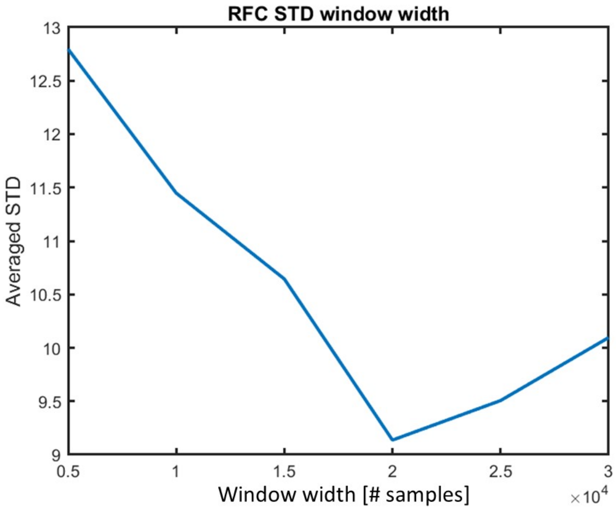

Figure 15. RFC standard deviation [%] results averaged along with the considered spectrum divisions.

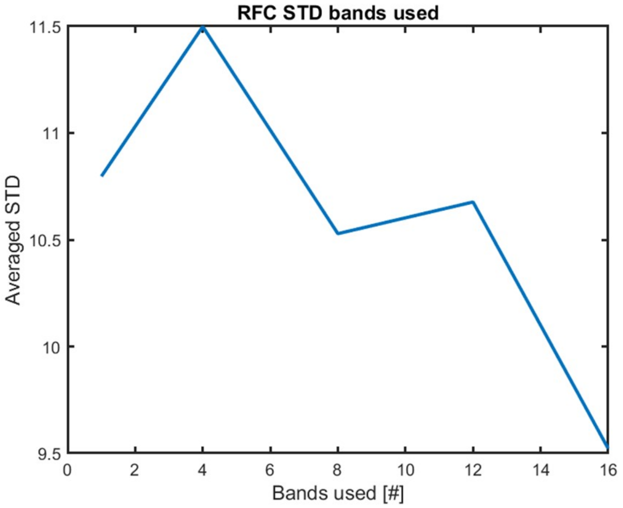

Figure 16. RFC standard deviation [%] averaged results along with the considered extraction window sizes.

There are variations in the resulted classification accuracy amongst the different applied extraction spectrum divisions and window width, see Figs. 5, 6, 7, 8, 9, 10, 11, 12, 13, 14, 15 and 16.

Cepstral coefficients analysis

We extracted cepstral coefficients from three acoustic signals associated with tectonic events with different moment magnitude (selected from the studied dataset), see Fig. 17. We consider that, for the studied range of moment magnitude, 12 coefficients can capture most of the information carried by the acoustic signals.

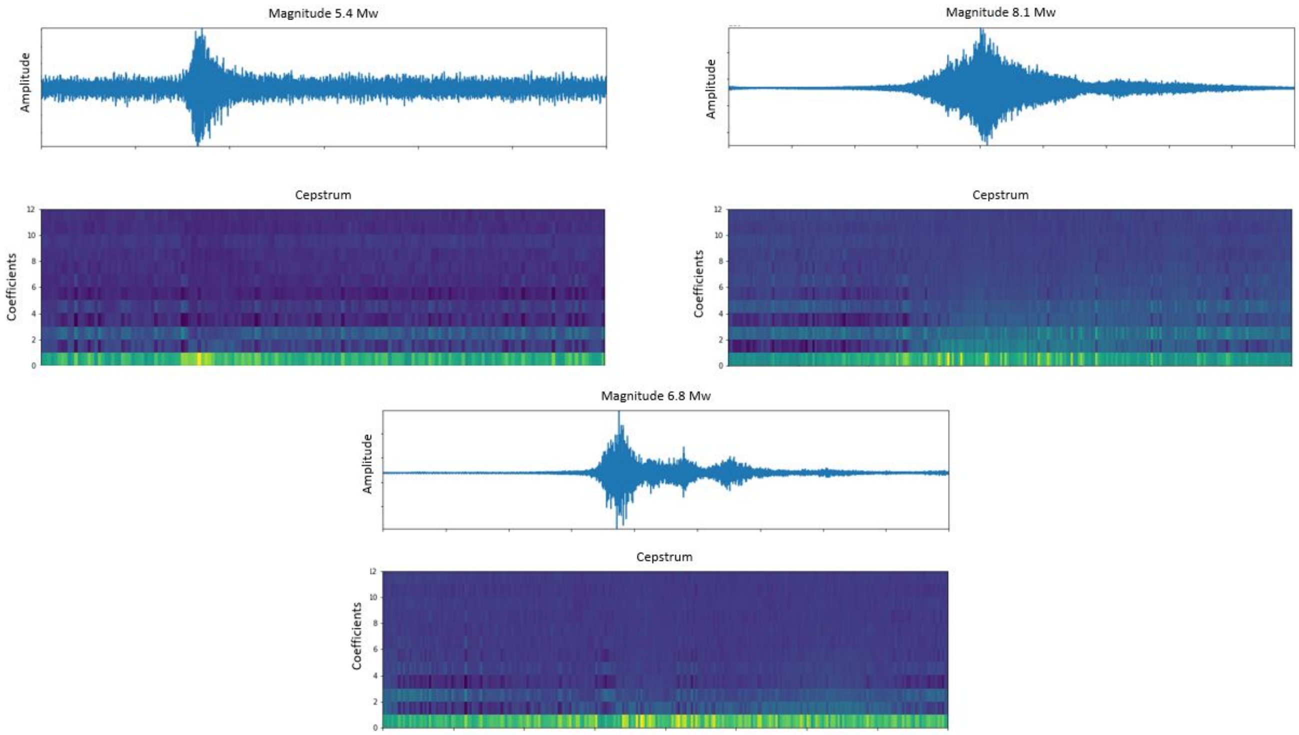

Figure 17. Cepstrum related to 5.4 M_w earthquake (12/15/2017); 6.8 M_w earthquake (28/02/2013); and 8.1 M_w earthquake (23/12/2004).

For the three studied earthquakes in this subsection, see Fig. 17, the highest coefficients lie on the first eight cepstral coefficient bands, carrying most of the information.

Wavelet transform parameters analysis

For binary classification and along with the introduced classification algorithms, we tested different types of discrete wavelets, wavelet orders and number of coefficient levels, see Tables 3, 4, 6 and 9.

Levels	Order 2	Order 5	Order 8
4	76.14 ± 7.58	72.64 ± 9.78	74.62 ± 8.80
6	79.12 ± 6.94	76.64 ± 8.86	78.14 ± 7.99
8	77.07 ± 6.87	75.64 ± 8.42	74.69 ± 10.10

Table 3. SVM accuracy [%] / ± Standard deviation [%] for wavelet transform features with Symlet wavelet, different wavelet orders and levels.

Levels	Order 2	Order 5	Order 8
4	74.14 ± 11.32	71.67 ± 8.56	75.62 ± 9.78
6	73.64 ± 10.93	78.10 ± 8.74	78.64 ±9.11
8	72.19 ± 7.53	76.12 ± 9.94	74.62 ±11.29

Table 4. RFC accuracy [%] / ± Standard deviation [%] for wavelet transform features with Symlet wavelet, different wavelet orders and levels.

Levels	Order 2	Order 5	Order 8
**4**	76.14 ± 7.58	72.64 ± 9.78	75.12 ± 9.49
**6**	79.12 ± 6.94	76.64 ± 8.58	75.67 ± 8.00
**8**	77.07 ± 6.87	74.62 ± 7.58	74.62 ± 7.91

Table 5. SVM accuracy [%] / ± Standard deviation [%] for wavelet transform features with Daubechies wavelet, different wavelet orders and levels.

Levels	Order 2	Order 5	Order 8
**4**	74.14 ± 11.32	70.14 ±8.95	76.12 ± 9.94
**6**	73.64 ± 10.93	76.12 ± 9.94	77.12 ± 8.10
**8**	72.19 ± 7.53	74.62 ± 9.87	76.62 ± 10.25

Table 6. RFC accuracy [%] / ± Standard deviation [%] for wavelet transform features with Daubechies wavelet, different wavelet orders and levels.

Regression accuracy distribution

The predicted values resulting from the application of the ML regression algorithms on the tested feature sets are plotted against the actual values to identify possible bias, see Figs. 18 and 19.

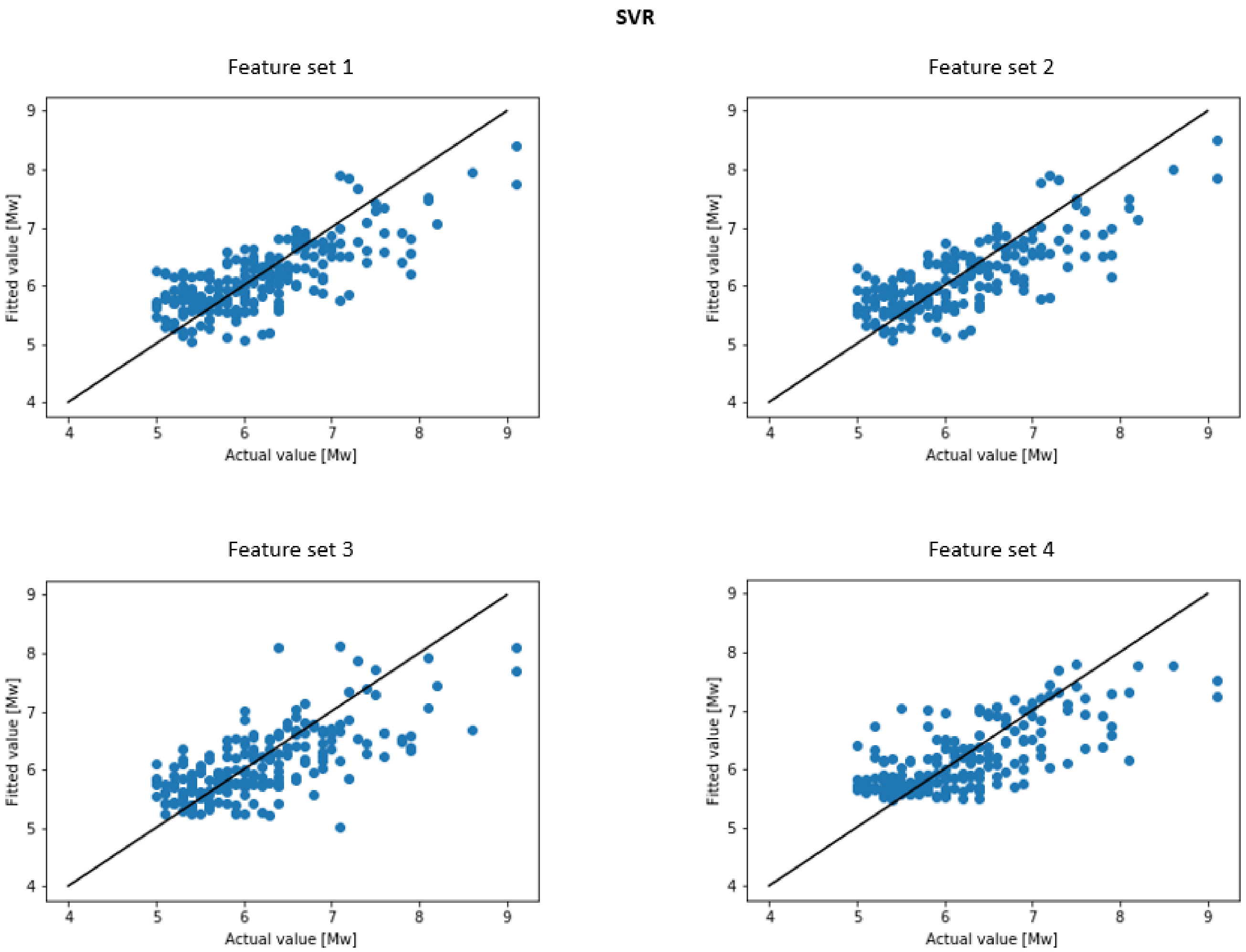

Figure 18. M_w values comparison between the actual values of the test set and the estimated values by SVR for the different feature sets.

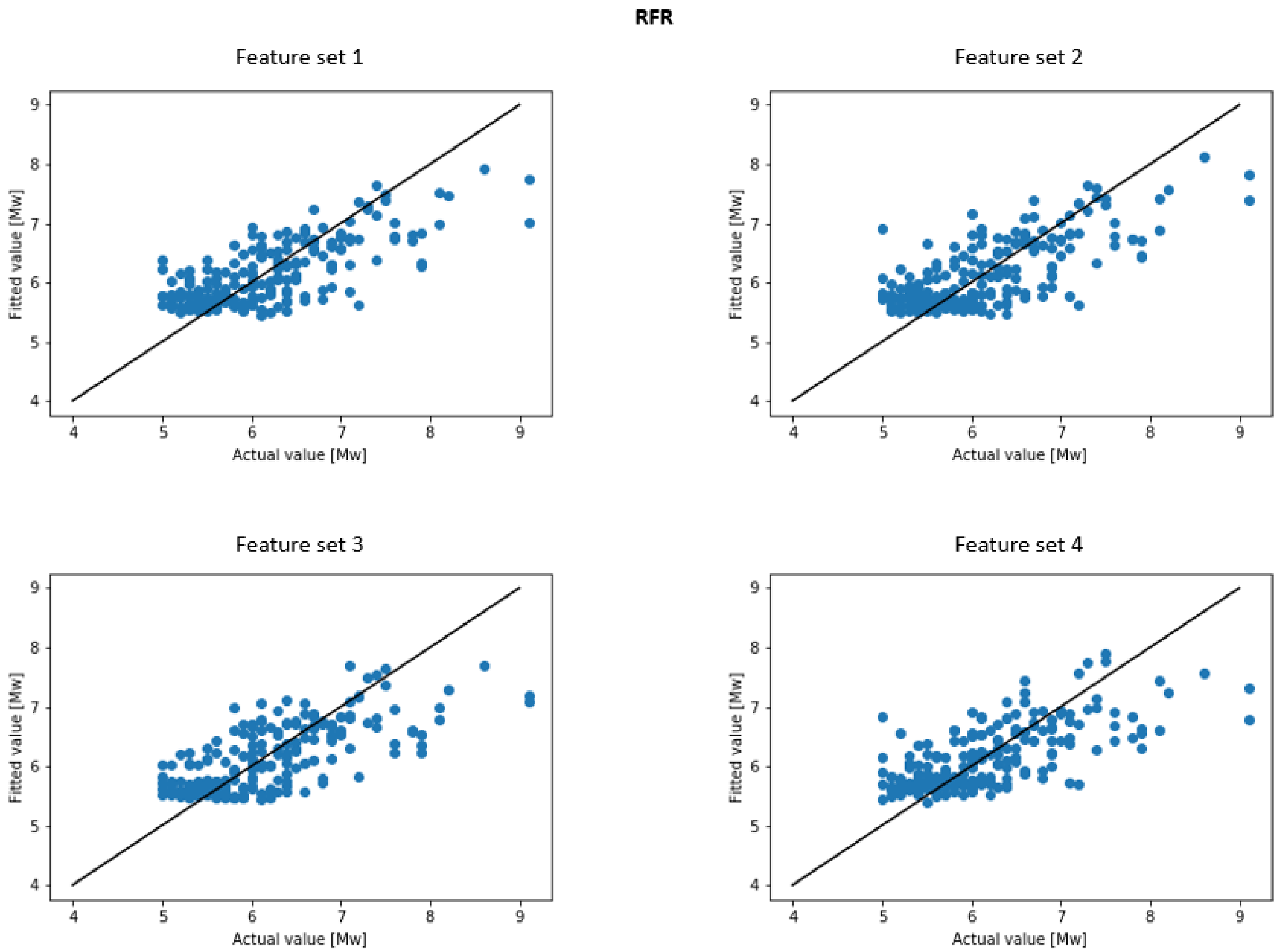

Figure 19. M_w values comparison between the actual values of the test set and the estimated values by RFR for the different feature sets.

In addition, the mean squared error for 10-fold is plotted for every moment magnitude bin.

	Magnitude [Mw]				
	**[5-6]**	**[6-7]**	**[7-8]**	**[8-9]**	**[9-10]**
**1st**	0.474	0.484	0.777	0.831	1.692
**2nd**	0.495	0.503	0.793	0.833	1.525
**3rd**	0.523	0.463	0.747	1.317	1.703
**4th**	0.514	0.456	0.869	1.063	2.058

Table 7. SVR mean squared errors for each considered feature set.

	Magnitude [Mw]				
	**[5-6]**	**[6-7]**	**[7-8]**	**[8-9]**	**[9-10]**
**1st**	0.487	0.434	0.831	0.78	1.073
**2nd**	0.492	0.433	0.88	0.858	0.48
**3rd**	0.501	0.434	0.793	1.12	1.852
**4th**	0.505	0.463	0.873	1.123	2.075

Table 8. RFR mean squared errors for each considered feature set.

Machine learning application on synthetic signals analysis

Synthetic signals were introduced and randomly shuffled into the dataset. Then, ML classification algorithms were applied with 10-fold validation technique and 5-fold grid search on the first three feature sets, see Figs. 20 and 21. Note that the synthetic signals added to the dataset are considered incoming from vertical motion slender faults.

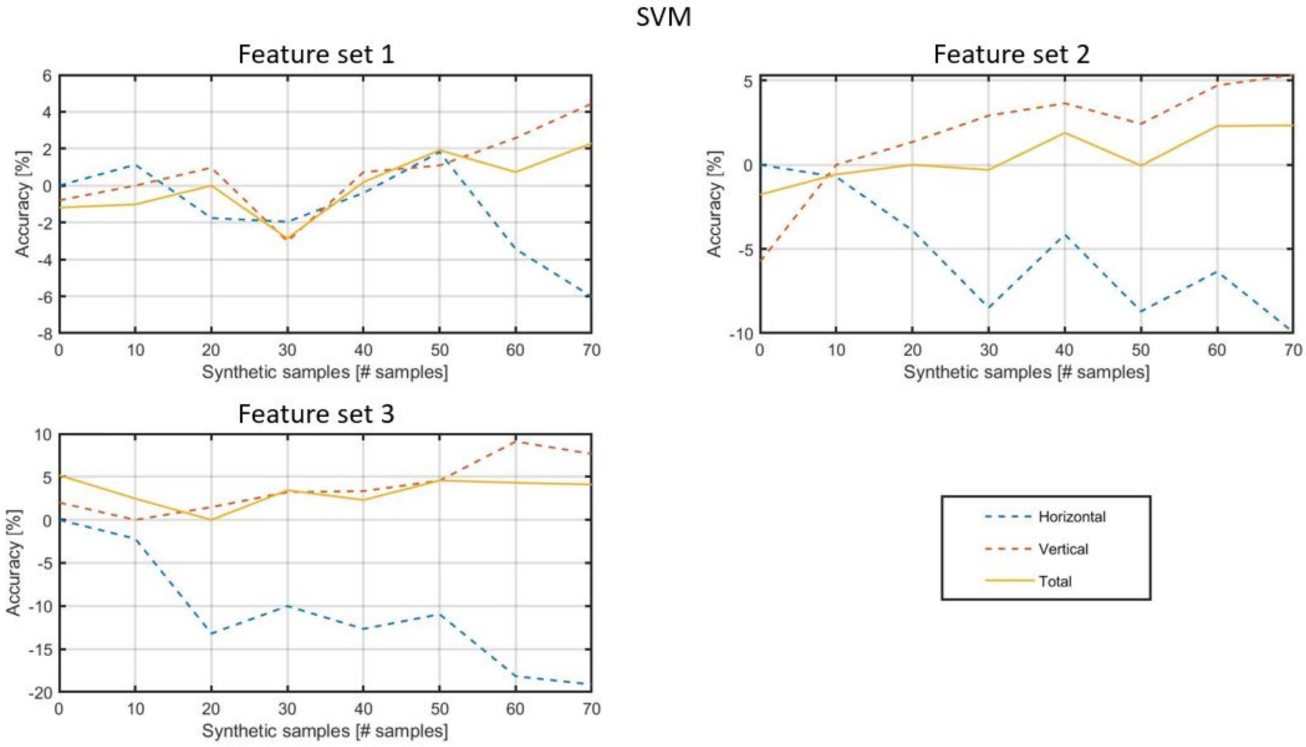

Figure 20. SVM accuracy [%], for three different feature sets and different amounts of added synthetic signals.

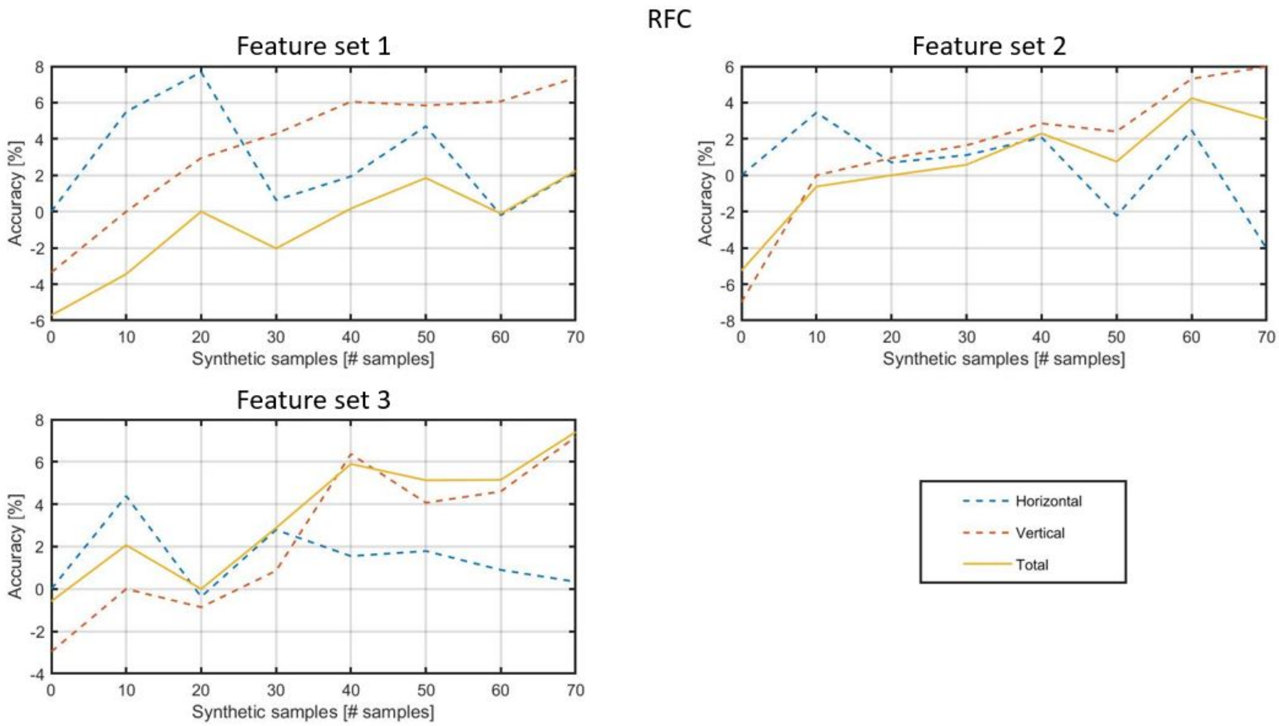

Figure 21. RFC accuracy [%], for three different feature approaches and different amounts of added synthetic signals.

List of earthquakes

List of earthquakes associated with the acoustic signals used in this project. The times are expressed as UTC (Coordinated Universal Time).

Table 9. In the column 'Type', '0' indicates strike-slip, '1' thrust and '2' normal event. In the column 'Tsunami', '0' indicates that no tsunami was recorded associated with the tectonic event and '1' indicates that there was a reported tsunami.

M_w	Date	Time	Location	Type	Tsunami
5	20/10/2008	15:51	Southeast of Honshu	0	0
5	02/01/2010	00:21	Mid-Indian ridge	0	0
5	25/01/2011	07:46	Andreanof islands, Aleutian islands	1	0
5	09/01/2013	05:27	Carlsberg ridge	2	0
5	08/04/2017	01:19	Macquarie island region	0	0
5.1	24/08/2008	01:00	Fiji islands region	0	0
5.1	22/02/2009	10:33	East of Kuril islands	2	0
5.1	16/01/2013	18:49	Southeast Indian ridge	0	0
5.1	07/02/2013	02:46	Sta. Cruz islands	2	0
5.1	09/01/2016	20:11	Western Indian-Antarctic ridge	0	0
5.1	17/05/2017	17:34	Macquarie island region	0	0
5.1	25/07/2017	21:12	West of Macquarie island	0	0
5.2	11/11/2008	12:18	Carlsberg ridge	2	0
5.2	16/12/2008	10:19	Mid-Indian ridge	0	0
5.2	17/12/2008	16:07	Southwest of Sumatra	1	0
5.2	01/01/2010	14:31	Mid-Indian ridge	0	0
5.2	31/08/2011	14:26	Mid-Indian ridge	0	0
5.2	15/01/2014	13:02	Mid-Indian ridge	0	0
5.2	26/09/2017	21:16	Southeast Indian ridge	0	0
5.3	05/01/2008	20:01	Near west coast of Sumatra	1	0
5.3	27/11/2008	09:17	Southeast Indian ridge	0	0

5.3	28/05/2010	18:32	Southern Sumatra	1	0
5.3	02/03/2012	10:11	Off coast of Honshu	1	0
5.3	29/07/2015	16:05	Fox Islands	1	0
5.3	04/08/2015	23:21	Kermadec islands region	1	0
5.3	03/09/2015	16:51	Off east coast of Honshu	2	0
5.3	17/01/2016	19:25	Mid-Indian ridge	0	0
5.3	08/10/2016	22:25	Tonga islands	0	0
5.3	03/04/2017	07:19	Mid-Indian ridge	0	0
5.4	14/01/2008	01:20	Indian ocean	2	0
5.4	01/02/2008	10:26	Tonga islands region	0	0
5.4	24/05/2009	07:03	Indian ocean	0	0
5.4	02/12/2010	23:39	Western Indian-Antarctic ridge	2	0
5.4	12/01/2011	13:05	West of Macquarie island	0	0
5.4	14/07/2012	14:22	Western Indian-Antarctic ridge	2	0
5.4	20/07/2015	05:28	Southwest Indian ridge	2	0
5.4	28/07/2015	21:21	Fox islands, Aleutian islands	1	0
5.4	27/10/2015	12:15	Mid-Indian ridge	0	0
5.4	29/01/2017	16:42	Southeast Indian ridge	2	0
5.4	09/05/2017	09:02	Andreanof islands, Aleutian islands	1	0
5.4	15/11/2017	00:23	Indian ocean triple junction	2	0
5.4	25/12/2017	12:02	Mid-Indian ridge	2	0
5.5	26/03/2008	18:33	Kuril islands	1	0
5.5	08/04/2008	02:09	South of Mariana islands	0	0
5.5	12/04/2008	08:50	Mid-Indian ridge	2	0
5.5	19/09/2008	22:49	Sta. Cruz islands region	2	0
5.5	25/02/2009	16:08	Southeast Indian ridge	0	0
5.5	13/01/2010	16:21	Tonga islands	0	0
5.5	07/01/2011	03:09	Off west coast of Sumatra	2	0
5.5	20/07/2012	03:40	Kuril islands	1	0
5.5	12/10/2013	20:01	Mid-Indian ridge	0	0
5.5	05/06/2015	14:54	Mid-Indian ridge	0	0
5.5	24/12/2017	17:33	Samoa islands region	2	0
5.6	07/01/2008	08:14	Aleutian islands region	1	0
5.6	11/03/2008	14:37	Fox islands, Aleutian islands	1	0
5.6	24/05/2009	06:49	Indian ocean	0	0
5.6	07/01/2010	08:29	Macquarie islands region	0	0
5.6	26/03/2012	16:58	Southwest Indian ridge	0	0
5.6	24/04/2012	09:50	Carlsberg ridge	2	0
5.6	06/11/2012	06:17	Carlsberg ridge	2	0
5.6	15/06/2014	18:19	Near east coast of Honshu	2	0
5.6	01/07/2015	14:30	Macquarie islands region	0	0
5.6	25/06/2017	03:01	Mid-Indian ridge	0	0
5.7	09/01/2009	03:44	Carlsberg ridge	0	0
5.7	30/01/2009	03:47	Tonga islands	0	0
5.7	26/03/2009	06:14	Mid-Indian ridge	2	0
5.7	15/04/2009	10:20	Owen fracture	0	0
5.7	05/07/2011	19:02	Fiji islands region	0	0
5.7	27/01/2013	09:59	Tonga islands region	2	0
5.7	04/04/2017	22:08	Andreanof islands, Aleutian islands	1	0
5.7	31/10/2017	04:58	Tonga islands region	2	0
5.7	22/04/2018	16:59	Tonga islands region	0	0
5.8	18/09/2008	11:58	near east coast of kamchatka	1	0
5.8	01/10/2009	06:13	Tonga islands region	1	0
5.8	29/04/2010	23:48	Western Indian-Antarctic ridge	0	0
5.8	14/12/2011	00:48	Tonga islands region	0	0

5.8	02/05/2012	12:18	West of Macquarie island	0	0
5.8	05/02/2014	20:52	Balleny islands region	0	0
5.8	01/06/2014	10:07	North Indian ocean	0	0
5.8	24/05/2017	16:36	Fox islands	2	0
5.8	21/04/2018	19:44	Western Indian-Antarctic ridge	2	0
5.9	27/06/2008	13:07	Andaman	2	0
5.9	21/12/2008	09:16	East coast of Honshu	2	0
5.9	19/03/2011	01:22	Near east coast of Honshu	1	0
5.9	11/05/2014	12:35	Southeast Indian ridge	0	0
5.9	29/06/2014	18:24	Tonga islands region	0	0
5.9	12/01/2016	09:45	Southeast Indian ridge	2	0
5.9	31/05/2016	10:04	Kuril islands region	1	0
5.9	14/10/2018	12:41	Southeast Indian ridge	0	0
6	22/01/2008	07:55	Tonga islands	0	0
6	13/01/2009	01:04	Mid-Indian ridge	0	0
6	23/09/2009	02:59	Macquarie island region	0	0
6	10/11/2009	02:48	Off southeast coast of India	0	0
6	09/06/2010	01:03	Tonga islands region	0	0
6	04/08/2010	23:48	East of Kuril islands	2	0
6	23/04/2011	10:12	Near east coast of Sonshu	1	0
6	23/05/2012	22:59	Western Indian-Antarctic ridge	0	0
6	25/11/2013	05:56	Kuril islands	1	0
6	28/10/2014	03:15	Tonga islands region	0	0
6	13/08/2015	10:39	Mid-Indian ridge	0	0
6	14/10/2015	05:43	East of Kuril islands	1	0
6	19/12/2015	02:10	Vanuatu islands	0	0
6	19/03/2017	15:43	Solomon islands	1	0
6.1	22/01/2008	10:49	Tonga islands region	0	0
6.1	02/11/2008	13:48	Aleutian islands region	1	0
6.1	21/04/2010	17:20	Tonga islands region	2	0
6.1	04/08/2011	13:51	Kuril islands	1	0
6.1	02/08/2012	09:56	New ireland	1	0
6.1	31/01/2013	03:33	Sta. Cruz islands	2	0
6.1	20/04/2013	13:12	Kuril islands region	1	0
6.1	08/12/2013	17:24	Kuril islands region	1	0
6.1	18/02/2015	09:32	Sta. Cruz islands region	0	0
6.1	24/03/2018	19:58	South east Indian ridge	0	0
6.2	07/09/2009	16:12	Java	2	0
6.2	01/02/2010	22:28	Solomon islands	1	0
6.2	05/02/2010	06:59	South east Indian ocean	0	0
6.2	17/08/2011	11:44	Off east coast of Honshu	2	0
6.2	23/09/2016	00:14	Off east coast of Honshu	1	0
6.2	20/09/2017	16:37	Off east coast of Honshu	2	0
6.3	09/08/2008	16:36	West of Macquarie island	0	0
6.3	10/07/2010	11:43	Mariana islands	0	0
6.3	14/08/2010	23:01	South of Mariana islands	2	0
6.3	03/09/2012	18:23	South of Java, Indonesia	2	0
6.3	20/07/2014	18:32	Kuril islands	1	0
6.3	03/11/2014	08:48	Mid-Indian ridge	0	0
6.3	27/08/2017	04:17	Admirality islands region	0	0
6.3	18/04/2019	14:46	Western Indian-Antarctic ridge	0	0
6.4	09/06/2004	22:52	Western Indian-Antarctic ridge	0	0
6.4	01/04/2009	03:55	New Guinea	0	0
6.4	13/10/2009	05:37	Fox islands, Aleutian islands	1	0
6.4	10/11/2010	04:05	Indian Ocean	0	0

6.4	22/03/2011	07:18	Off east coast of Honshu	2	0
6.4	31/03/2011	00:12	Fiji islands region	0	0
6.4	22/09/2011	23:07	Tonga islands	0	0
6.4	04/10/2013	17:26	Mid-indian ridge	0	0
6.4	09/11/2015	16:03	South Alaska	1	0
6.4	08/02/2016	16:19	Solomon islands	1	0
6.4	13/08/2017	03:08	Indonesia	1	0
6.5	03/03/2008	09:31	Kuril islands region	1	0
6.5	03/09/2010	11:16	Andreanof islands, Aleutian islands	1	0
6.5	12/03/2011	01:47	Off east coast of Honshu	2	0
6.5	16/11/2012	18:12	Kuril islands region	1	0
6.5	04/09/2013	02:32	Andreanof islands, Aleutian islands	1	0
6.5	11/07/2014	19:22	Off east coast of Honshu	2	1
6.6	18/04/2009	19:18	Kuril islands region	1	0
6.6	12/08/2009	22:48	Near east coast of Honshu	1	0
6.6	31/07/2011	23:39	Near north coast of New Guinea	0	0
6.6	26/07/2012	05:33	Mauritius	0	0
6.6	09/10/2012	12:32	West of Macquarie island	0	0
6.6	29/06/2014	17:15	Samoa islands region	0	0
6.6	15/08/2018	21:56	Andreanof islands, Aleutian islands	1	0
6.7	01/01/2005	06:25	Off west coast of northern Sumatra	0	0
6.7	09/05/2008	21:51	South of Mariana islands	2	0
6.7	17/08/2009	00:05	Southwestern Ryukyu islands	0	0
6.7	22/06/2011	21:50	Off east coast of Honshu	1	0
6.7	16/09/2011	19:26	Near east coast of Honshu	1	0
6.7	28/04/2012	10:08	Tonga islands region	2	0
6.8	28/02/2013	14:05	Kuril islands	1	0
6.8	03/08/2014	00:22	Caroline islands	0	0
6.8	17/09/2014	06:14	Mariana islands	2	0
6.8	22/05/2015	23:59	Solomon islands	0	0
6.9	30/01/2007	04:54	West of Macquarie island	0	0
6.9	07/04/2009	04:23	Kuril islands region	1	0
6.9	13/08/2010	21:19	South Mariana islands	2	1
6.9	14/03/2012	09:08	Off east coast of Honshu	2	1
6.9	18/07/2015	02:27	Solomon islands	2	1
6.9	27/07/2015	04:49	Fox islands, Aleutian islands	1	1
6.9	03/01/2017	21:52	South Fiji islands	2	1
7	10/07/2011	00:57	Off east coast of Honshu	0	1
7	06/02/2013	01:54	Sta. Cruz islands	0	0
7	08/02/2013	15:26	Sta. Cruz islands	0	1
7	30/08/2013	16:25	Andreanof islands, Aleutian islands	1	0
7.1	22/11/2004	20:26	Off west coast of Stewart island	1	1
7.1	12/04/2008	00:30	Macquarie island region	1	0
7.1	06/02/2013	01:23	Sta. Cruz Islands	2	0
7.1	25/10/2013	17:10	Off east coast of Honshu	2	1
7.1	04/12/2015	22:25	Southeast Indian Ridge	2	0
7.2	26/12/2004	04:21	Nicobar islands, India region	1	0
7.2	10/01/2012	18:37	Off west coast of northern Sumatra	0	0
7.2	12/08/2016	01:26	Loyality islands	0	1
7.3	09/03/2011	02:45	Off east coast of Honshu	1	0
7.3	19/04/2013	03:06	Kuril islands region	2	1
7.4	15/01/2009	17:49	Kuril islands region	1	1
7.4	07/10/2009	23:14	Vanuatu islands	1	0
7.4	21/12/2010	17:19	Bonin islands, Japan region	2	1
7.5	10/08/2009	19:56	Andaman	2	1

7.5	12/06/2010	19:27	Indian Ocean	1	1
7.6	19/03/2009	18:17	Tonga islands region	1	1
7.6	07/10/2009	22:03	Vanuatu islands	1	1
7.6	31/08/2012	12:47	Philippine islands region	1	1
7.8	07/10/2009	22:19	Sta. Cruz islands	1	0
7.8	02/03/2016	12:49	Sumatra	0	0
7.9	06/02/2013	01:12	Sta. Cruz islands	1	1
7.9	30/05/2015	11:23	Bonin islands, Japan Region	2	0
7.9	17/12/2016	10:51	New Ireland	1	1
8.1	23/12/2004	14:59	North of Macquarie Island	0	1
8.1	29/09/2009	17:48	Sumatra	2	1
8.2	11/04/2012	10:43	Sumatra	0	1
8.6	11/04/2012	08:39	Sumatra	0	1
9.1	26/12/2004	01:01	Sumatra	1	1
9.1	11/03/2011	06:15	Tohoku	1	1
